# *MFN2* knockdown promotes osteogenic differentiation of iPSC-MSCs through aerobic glycolysis mediated by the Wnt/β-catenin signaling pathway

**DOI:** 10.1186/s13287-022-02836-w

**Published:** 2022-04-12

**Authors:** Lidi Deng, Siqi Yi, Xiaohui Yin, Yang Li, Qingxian Luan

**Affiliations:** 1 Department of Periodontology, Peking University School and Hospital of Stomatology & National Center of Stomatology & National Clinical Research Center for Oral Diseases & National Engineering Laboratory for Digital and Material Technology of Stomatology & Beijing Key Laboratory of Digital Stomatology & Research Center of Engineering and Technology for Computerized Dentistry Ministry of Health & NMPA Key Laboratory for Dental Materials, No. 22, Zhongguancun South Avenue, Haidian District, Beijing, 100081 People’s Republic of China; 2 Department of Cell Biology, School of Basic Medical Sciences, Peking University Stem Cell Research Center, Peking University, Beijing, 100191 People’s Republic of China; 3 Department of First Clinical Division, Peking University School and Hospital of Stomatology & National Center of Stomatology & National Clinical Research Center for Oral Diseases & National Engineering Laboratory for Digital and Material Technology of Stomatology & Beijing Key Laboratory of Digital Stomatology & Research Center of Engineering and Technology for Computerized Dentistry Ministry of Health & NMPA Key Laboratory for Dental Materials, No. 22, Zhongguancun South Avenue, Haidian District, Beijing, 100081 People’s Republic of China

**Keywords:** Mitofusin-2, Induced pluripotent stem cells, Mesenchymal stem cells, Wnt/β-catenin signaling pathway, Aerobic glycolysis, Osteogenic differentiation

## Abstract

**Background:**

Mitofusin-2 (*MFN2*) is a kind of GTPase that participates in the regulation of mitochondrial fusion, which is related to a variety of physiological and pathological processes, including energy metabolism, cell differentiation, and embryonic development. However, it remains unclear whether *MFN2* is involved in the metabolism and osteogenic differentiation of mesenchymal stem cells (MSCs).

**Methods:**

*MFN2* knockdown (*MFN2*-KD) and *MFN2*-overexpressing (*MFN2*-OE) induced pluripotent stem cell-derived mesenchymal stem cells (iPSC-MSCs) were constructed by lentivirus. The commercial kits were utilized to detect the glycolysis and oxidative phosphorylation (OXPHOS) rate. Flow cytometry, Western blot, quantitative real-time polymerase chain reaction (qRT-PCR), RNA-seq, immunofluorescence, and immunoprecipitation were employed for phenotype and molecular mechanism assessment.

**Results:**

We demonstrated that *MFN2* and Wnt/β-catenin signaling pathway regulated glycolysis of iPSC-MSCs. The lack of *MFN2* promoted the osteogenic differentiation of iPSC-MSCs, and aerobic glycolysis in the presence of sufficient oxygen, which increased glucose consumption and lactic acid production, as well as the glycolytic enzyme activity and gene expression. Inhibiting the Wnt/β-catenin signaling pathway normalized the enhanced glycolytic rate and osteogenic differentiation of *MFN2*-KD iPSC-MSCs. *MFN2*-OE iPSC-MSCs displayed the opposite phenotype.

**Conclusions:**

Downregulating *MFN2* promotes osteogenic differentiation of iPSC-MSCs through aerobic glycolysis mediated by the Wnt/β-catenin signaling pathway. Our research reveals the new function of *MFN2* in regulating the osteogenic differentiation and energy metabolism of MSCs, which will provide a new therapeutic target and theoretical basis for alveolar bone repair and periodontal regenerative treatment.

**Supplementary Information:**

The online version contains supplementary material available at 10.1186/s13287-022-02836-w.

## Introduction

Periodontal regeneration treatment has been a hot spot in the field of periodontal research in recent years. Mesenchymal stem cells (MSCs) are considered to be the most promising resource in periodontal tissue regeneration [1]. The number of autologous MSCs is limited, and our previous study reprogrammed human gingival fibroblasts into iPSCs, which is an endless source of induced pluripotent stem cell-derived mesenchymal stem cells (iPSC-MSCs). Understanding the molecular mechanism of the osteogenic differentiation process and promoting the osteogenic differentiation efficiency of MSCs are very important for periodontal regeneration.

Aerobic glycolysis and oxidative phosphorylation (OXPHOS) are utilized during the proliferation of MSCs [2]. Some types of stem cells and rapidly proliferating cells such as cancer cells consume glucose mainly through glycolysis, rather than OXPHOS when oxygen is sufficient. This phenomenon of cellular aerobic glycolysis is similar to the Warburg effect [3]. Previous studies have found that the Warburg effect is critical for bone formation, which chooses glycolysis rather than OXPHOS as the main metabolic mode. It consumes a large amount of glucose and produces lactic acid as the main end product [4]. Another study demonstrates that MSCs make more use of OXPHOS in osteogenic culture conditions [5]. It is worth noting that it remains controversial about the main pathway of energy metabolism and its molecular mechanism of regulation during the osteogenic differentiation of MSCs.

Mitofusin 2 (*MFN2*) is encoded by the *MFN2* gene located on chromosome 1 in the nuclear genome [6]. MFN2 is a multifunctional protein located on the mitochondrial outer membrane; in addition to mitochondrial fusion, it participates in various biological processes under physiological and pathological conditions, such as cell proliferation, autophagy, apoptosis, endoplasmic reticulum-mitochondrial connection, and stem cell differentiation [7]. Our previous studies showed that *MFN2* knockdown promoted neural ectodermal differentiation of hESCs [8]. Several studies have shown that it also plays an important role in mitochondrial metabolism and function, mainly manifested in the effect on glucose oxidation and cellular respiration [9]. Studies have shown that the lack of *MFN2* inhibits the expression of OXPHOS complexes, and its participation in mitochondrial metabolism is independent of mitochondrial fusion [10]. However, *MFN2* knockdown significantly increases the expression of genes encoding glycolytic enzymes and glycolytic metabolites [11]. In the early stage of osteogenesis, the lack of *Mfn2* enhances the osteogenic differentiation and cortical bone accumulation of mice in vitro and in vivo [12]. These findings indicate that *MFN2* ablation may promote the conversion of glycolytic bioenergy to meet the energy requirements of specific cells such as osteoblasts.

Studies have found that the Wnt/β-catenin signaling pathway stimulates glycolysis by upregulating the expression of downstream glycolytic enzymes hexokinase-2 (HK2), lactate dehydrogenase A (LDHA), and pyruvate dehydrogenase kinase 1 (PDK1) [13, 14]. Wnt/β-catenin pathway stimulates EGFR and downstream PI3K/Akt pathway and hypoxia-inducible factor 1α (HIF-1α), thereby indirectly activating aerobic glycolysis [15]. *MFN2* expression correlates with the inactivation of the Wnt/β-catenin signaling pathway. Furthermore, MFN2 ablation activates downstream genes including cyclin D1, c-Myc, c-Jun, and Axin2 [16]. However, it remains unclear whether *MFN2* is involved in the osteogenic differentiation and regulation of energy metabolism in MSCs.

Here we reported that *MFN2* ablation promoted the osteogenic differentiation of iPSC-MSCs. *MFN2* ablation increased glucose consumption and lactic acid production in the presence of sufficient oxygen, as well as the expression of glycolytic enzymes, which promotes the conversion of cell metabolism to aerobic glycolysis. Inhibiting the Wnt/β-catenin signaling pathway normalized the enhanced glycolysis and osteogenic differentiation of *MFN2*-KD iPSC-MSCs. *MFN2* overexpressing iPSC-MSCs displayed the opposite phenotype. Our results reveal the new role of *MFN2* in the osteogenic differentiation and energy metabolism of MSCs, which are expected to provide new ideas for alveolar bone repair and periodontal regeneration.

## Materials and methods

### Cell culture

iPSCs were reprogrammed from human gingival fibroblasts in our previous studies [17]. According to the manufacturer’s instructions, from day 1–3, iPSCs were cultured by STEMdiff™-ACF mesenchymal induction medium (STEMCELL Technologies, Canada) and MesenCult™-ACF Plus medium (STEMCELL Technologies, Canada) from day 4–5. From day 6 to day 21, cells were passaged at approximately 80% confluent and cultured with MesenCult™-ACF Plus medium to get iPSC-MSCs. PDLSCs were cultured in DMEM containing 10% FBS (Gibco, USA) and 1% penicillin–streptomycin (Gibco, USA). Cells were cultured in a cell culture incubator (Thermo Fisher Scientific, USA) containing 5% CO_2_ at 37 °C.

### Flow cytometry

Cells were digested into single-cell suspension by trypsin (Gibco, USA) and stained with antibody for 30 min at 4 °C. Cells were then analyzed by CytoFLEX S flow cytometer (Beckman Coulter, USA) and Cytexpert software (ver. 2.3, Beckman Coulter, USA). The antibodies were as follows: CD73-FITC (eBioscience, USA), CD90-FITC (BioLegend, USA), CD105-FITC (BioLegend, USA), CD34-PE (BD Biosciences, USA), and CD45-APC-A750 (eBioscience, USA).

### Construction of *MFN2 *knockdown (*MFN2*-KD) iPSC-MSCs by lentivirus

A pair of oligonucleotides encoding shRNA targeting against *MFN2* (GGAAGAGCACCGTGATCAATG) and NC (negative control, TTCTCCGAACGTGTCACGT) were cloned into GV307 plasmid (Genechem, China) with XhoI and EcoRI restriction sites. The plasmid was transformed to competent cells and screened by ampicillin-containing agar plates. The PCR product was identified and sequenced. pGag/Pol, pRev, pVSV-G, and transfection reagent (Genechem, China) were utilized to package the lentiviral particles and then infected the 293 T cells for 6 h. After being cultured for 48 h, the virus was concentrated with ultracentrifuge (Beckman Coulter, USA). For infection, fresh MesenCult™-ACF Plus medium containing 10 µl concentrated virus (10^8^ TU/ml) and 8 μg/ml polybrene was added to the iPSC-MSCs with 50% confluence for 12 h. The positive cells were screened with 0.3 µg/ml puromycin (Thermo Fisher Scientific, USA) 48 h after infection.

### Construction of *MFN2* overexpressing (*MFN2*-OE) iPSC-MSCs by lentivirus

The linearized GV492 vector (Genechem, China) was obtained by digestion with BamHI and AgeI. The target gene fragment was amplified by PCR (primers with underlined homologous recombination sequence: *MFN2*-1: AGGTCGACTCTAGAGGATCCCGCCACCATGTCCCTGCTCTTCTCTCG; and *MFN2*-2: TCCTTGTAGTCCATACCTCTGCTGGGCTGCAGGTACTGGTG). The linearized vector and target gene fragment were connected. Empty vector was utilized as negative control (NC). The following steps transformation, virus packaging, purification, and infection were the same as mentioned before.

### Transmission electron microscopy

The iPSC-MSCs were fixed in 3% glutaraldehyde overnight, rinsed with PBS, and fixed in 1% osmium tetroxide for 3 h. Then the cells were dehydrated in gradient ethanol and acetone, embedded in epoxy resin and sliced. Images were obtained by an electron microscope (JEOL, Japan).

### Cell counting kit-8 (CCK-8) assay

Cells were seeded in a 96-well plate with a density of 2000 cells/well, and 3 parallel wells were set for each group at each time point. After 0, 2, 4, 6, 8 days of culture, cells were added with 10 µl CCK-8 reagent (Dojindo, Japan), 100 µl DMEM, and incubated for 1 h at 37 °C. A microplate reader (Molecular Devices, USA) was used to measure the absorbance at 450 nm.

### Cell cycle assay

The cells were trypsinized, centrifuged at 1000*g* for 3 min, rinsed with precooled PBS and centrifuged again, fixed in 70% precooled ethanol at 4 °C overnight. The cells were centrifuged again, washed twice with precooled PBS, incubated with RNase A and PI (Beyotime, China) at 37 °C in the dark for 30 min, and the cell cycle (DNA content) was detected by flow cytometry (BD, USA) and evaluated by the ModFit software.

### Trilineage differentiation of MSCs

For the osteogenic differentiation, the induction medium (IM) included DMEM supplemented with 10% FBS (Gibco, USA), 50 µM vitamin C (Sigma-Aldrich, USA), 10 mM sodium β-glycerophosphate (Sigma-Aldrich, USA), and 0.1 µM dexamethasone (Sigma-Aldrich, USA). iPSC-MSCs were fixed with 95% ethanol after 14 days of differentiation and stained with Alizarin red staining solution (Solarbio, China).

For adipogenic differentiation, cells were cultured in DMEM with 10% FBS, 0.5 µM IBMX (Sigma-Aldrich, USA), 200 µM indomethacin (Sigma-Aldrich, USA), 10 µM insulin (Sigma-Aldrich, USA), and 1  µM dexamethasone. Cells were stained with Oil Red O staining solution (Solarbio, China).

For chondrogenic differentiation, cells were cultured in DMEM with 10% FBS, 10 ng/ml TGF-β1 (R&D Systems, USA), 40 µg/ml proline (Sigma-Aldrich, USA), 100 µg/ml sodium pyruvate (Sigma-Aldrich, USA), 50 µg/ml vitamin C, and 0.1 µM dexamethasone. Cells were stained with Alcian blue staining solution (Solarbio, China).

### Quantitative real-time polymerase chain reaction (qRT-PCR) analysis

Total RNA was extracted by Trizol reagent (Thermo Fisher Scientific, USA), and reverse transcription was performed using PrimeScript RT kit (Takara, Japan) according to the manufacturer’s instructions. qRT-PCR was conducted using Power Up SYBR Green Master Mix (Thermo Fisher Scientific, USA) in CFX384 Touch Real-Time PCR Detection System (Bio-Rad, USA). β-Actin was employed as an internal control. The primer sequences are shown in Table 1.

**Table 1:** Primer sequences used for qRT-PCR

Gene	Forward primer	Reverse primer
*MFN2*	CACAAGGTGAGTGAGCGTCT	CGTTGAGCACCTCCTTAGCA
*RUNX2*	TCTAAATCGCCAGGCTTCAT	GAGGACCTACTCCCAAAGGA
*OSX (SP7)*	TCCCTGCTTGAGGAGGAAG	TAGCATAGCCTGAGGTGGGT
*ALP*	CCACGTCTTCACATTTGGTG	AGACTGCGCCTGGTAGTTGT
*OCN*	ATGAGAGCCCTCACACTCCT	CTTGGACACAAAGGCTGCAC
*HK2*	GTGAATCGGAGAGGTCCCAC	CAAGCAGATGCGAGGCAATC
*LDHA*	GCCGATTCCGGATCTCATTG	CCAGCCTTTCCCCCATTAGG
*PDK1*	AGGCGTTGCAAGTATCACCA	CACACCAAAGCAGGAAAGGC
*HIF1A*	TCAAAGTCGGACAGCCTCAC	ATCCATTGATTGCCCCAGCA
*ACTB*	CTCGCCTTTGCCGATCC	TCTCCATGTCGTCCCAGTTG

### Western blot analysis

Total protein was extracted in RIPA lysis and extraction buffer (Thermo Fisher Scientific, USA) and separated by sodium dodecyl sulfate–polyacrylamide gel electrophoresis (SDS-PAGE). The separated proteins were transferred to a polyvinylidene difluoride (PVDF) membrane (Merck Millipore, USA). Membranes were incubated by primary and secondary antibodies after blocking in 5% skim milk. The results were visualized by Immobilon ECL Ultra Western HRP substrate (Merck Millipore, USA). The antibodies were as follows: VDAC (1∶1000, Proteintech, USA), MFN2 (1∶1000, Abcam, USA), RUNX2 (1∶1000, Proteintech, USA), OSX (1∶1000, Abcam, USA), ALP (1∶1000, Abcam, USA), OCN (1∶1000, Proteintech, USA), HIF-1α (1∶1000, Proteintech, USA), and β-catenin (1∶1000, Proteintech, USA).

### RNA-sequencing (RNA-seq)

Total RNA of the WT and *MFN2*-KD iPSC-MSCs were collected. Magnetic beads with polythymine were used to enrich mRNA containing polyA tails. The enriched mRNA was fragmented and reverse-transcribed into double-stranded cDNA. The ligation product was amplified by PCR and heat-denatured to obtain a single-stranded product, which is circularized to obtain a DNA library. The library preparations were sequenced on the BGISEQ-500 platform after quality inspection. Raw reads obtained by sequencing were filtered to get clean reads, which were compared to reference genes. mRNA quantification and differential expression analysis were performed, and *P*-value < 0.05 was considered differentially expressed. Kyoto Encyclopedia of Genes and Genomes (KEGG) pathway and Gene Ontology (GO) enrichment analysis on differentially expressed genes were performed on the Multi-omics System of BGI Genomics. In short, for GO analysis, all candidate differentially expressed genes were mapped and calculated to the GO database. A hypergeometric test was used to find out significantly enriched GO terms compared to the background genes. For KEGG pathway analysis, a hypergeometric test was used to find out significantly enriched pathways compared with background genes. The results were corrected by multiple tests to obtain Q value, and Q value ≤ 0.05 was defined as significantly enriched.

### Glycolysis (extracellular acidification) assay and extracellular oxygen consumption assay

According to the manufacturer's protocols, cells were seeded on a 96-well plate (5 × 10^4^ cells/well). The glycolysis (basal glycolysis and glycolytic capacity) rate was measured by fluorescence intensity in a fluorescence microplate reader (Molecular Devices, USA) with Glycolysis Stress Test Complete Assay Kit (Abcam, USA). Extracellular oxygen consumption assay was performed using Mitochondrial Stress Test Complete Assay Kit (Abcam, USA). Basal glycolysis rate refers to the increase in extracellular acidification caused by the proton output of glycolysis. Glycolytic capacity refers to the maximum glycolytic capacity of cells while OXPHOS is blocked by oligomycin. Basal respiration rate refers to the oxygen consumption used to meet mitochondrial proton leakage and cellular ATP demand, indicating the energy demand under basal conditions. Maximal respiration rate refers to the stimulation of the mitochondrial respiratory chain to operate to the maximum extent after FCCP treatment, indicating the maximum respiration potential of the cell.

### HK (hexokinase) and LDH (lactate dehydrogenase) activity assay

Cells were harvested, and the activity of HK and LDH was performed by Hexokinase Activity Assay Kit (Abcam, USA) and Lactate Dehydrogenase Assay Kit (Abcam, USA) according to the manufacturer's protocols. In brief, the cells were collected by trypsin and washed 3 times with precooled PBS, resuspended in precooled assay buffer, centrifuged at 12,000 rpm at 4 °C for 5 min to remove insoluble materials. The supernatant was collected, mixed with the reaction mixture, and incubated at room temperature in the dark for 40 min. The absorbance at 450 nm was measured by the microplate reader (BD, USA) and calculated the relative activity.

### Lactic acid measurements and glucose consumption assay

According to the manufacturer's protocols, for lactic acid measurement, cells were lysed and assayed with the Lactic Acid Assay Kit (Nanjing Jiancheng, China). In brief, the cells were lysed by ultrasonic on ice, and the enzyme working solution and the color reagent were added and mixed. The absorbance at 530 nm was measured by the microplate reader (BD, USA), and the lactic acid concentration was calculated according to the standard curve.

For glucose consumption measurement, the media cultured cells for 24 h were assayed using Glucose (HK) Assay Kit (Sigma-Aldrich, USA). In brief, the same amount of cells was seeded in the well plate, and the glucose determination reagent was added. The absorbance at 340 nm was measured by the microplate reader (BD, USA). The standardized glucose concentration and consumption were calculated based on the glucose standard solution and the amount of sample protein.

### Immunofluorescence

Cells were fixed in 4% paraformaldehyde for 30 min, permeabilized in 0.3% Triton X-100 (Sigma-Aldrich, USA) for 20 min, and blocked with 5% bovine serum albumin (BSA) (Sigma-Aldrich, USA) for an hour. Cells were incubated with primary antibodies for an hour and secondary antibody DyLight 488 (ZSGB-Bio, China) for an hour. The nucleus was stained by DAPI (Thermo Fisher Scientific, USA). Images were acquired with a confocal microscope (Leica, Germany). The primary antibodies were as follows: OCT4 (1∶100, Santa Cruz Biotechnology, USA) and NANOG (1∶100, Abcam, USA).

### Immunoprecipitation (IP)

Immunoprecipitation was carried out by Immunoprecipitation Kit (Beyotime, China) according to the manufacturer's protocol. In brief, cells were lysed in prechilled cell lysate and centrifuged to remove cell debris. One part is used as input, and the other part is incubated with protein A/G magnetic beads bound to MFN2 antibody (1∶50, CST, USA) or IgG at 4 °C overnight. Input and proteins eluted from protein A/G magnetic beads were detected by Western blot.

### Statistical analysis

All experiments were performed three times independently. The biological triplicate results were presented as means ± standard deviation using GraphPad Prism (ver. 6.01, GraphPad Software Inc, USA). The data were analyzed by one-way ANOVA for comparisons. *P* < 0.05 was considered statistically significant.

## Results

### *MFN2* knockdown promotes osteogenic differentiation of iPSC-MSCs

**Fig. 1:**
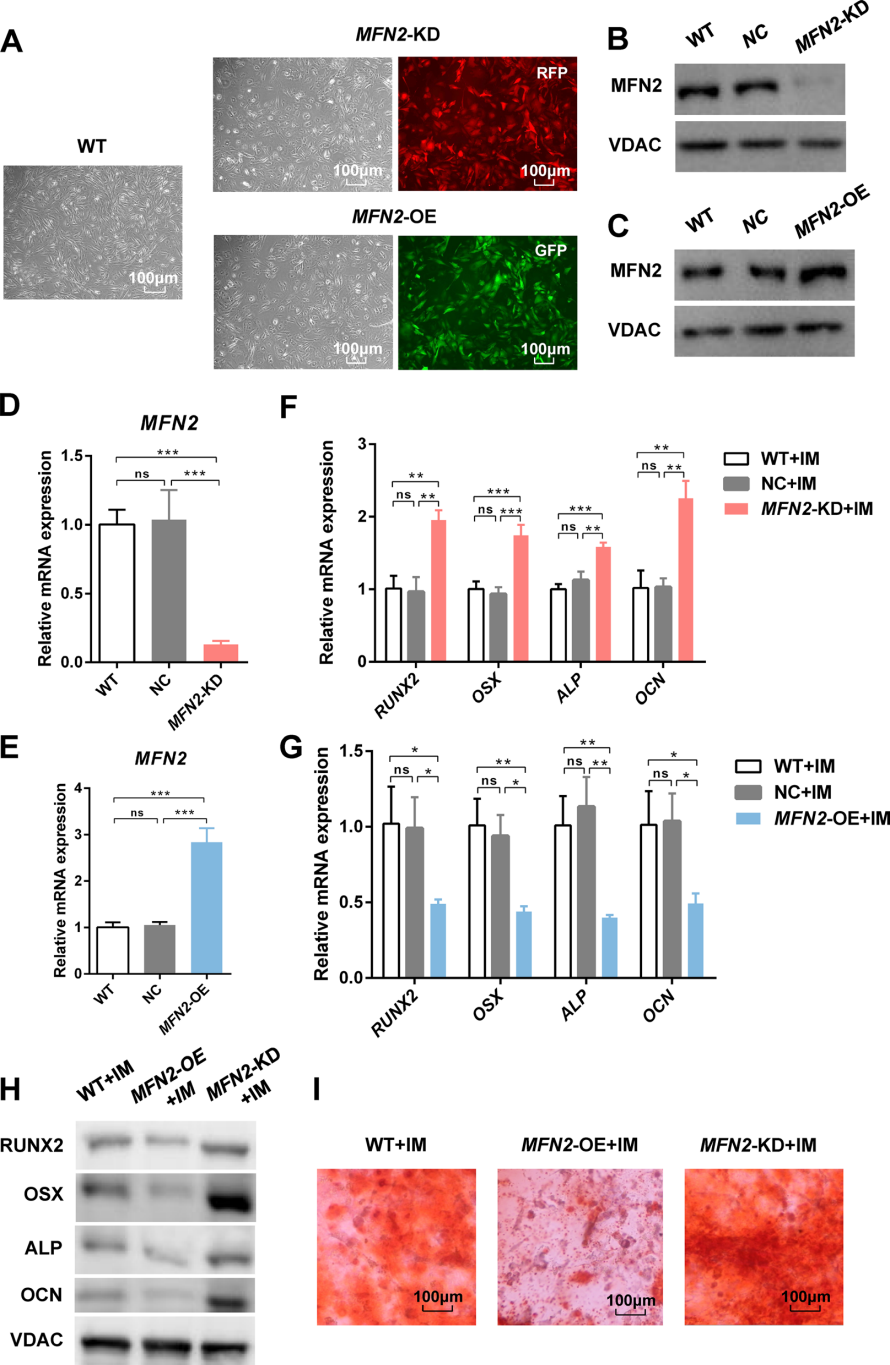
*MFN2* knockdown promotes osteogenic differentiation of iPSC-MSCs. **A** The morphology of WT, *MFN2*-KD, and *MFN2*-OE iPSC-MSCs under optical and fluorescence microscope. **B**–**C** Western blot analysis of MFN2 in WT, *MFN2*-KD, and *MFN2*-OE iPSC-MSCs. **D**–**E** qRT-PCR analysis of *MFN2* in WT, *MFN2*-KD, and *MFN2*-OE iPSC-MSCs. **F**–**H** qRT-PCR and Western blot analysis of osteogenic differentiation markers in WT, *MFN2*-KD, and *MFN2*-OE iPSC-MSCs after osteogenic differentiation. **I** Alizarin red staining of WT, *MFN2*-KD, and *MFN2*-OE iPSC-MSCs after osteogenic differentiation. RFP (red fluorescent protein) and GFP (green fluorescent protein) are the label proteins of lentivirus. NC: negative control. IM, osteogenic induction medium. ns, no significant. Data are expressed as mean ± standard deviation (SD), *P < 0.05, **P < 0.01, ***P < 0.001

iPSCs were reprogrammed from human gingival fibroblasts in our previous studies, which highly expressed pluripotency markers (Additional file 1: Fig. S1). *MFN2*-KD and *MFN2*-OE iPSC-MSCs showed little change in morphology with the wild type (WT) iPSC-MSCs (Fig. 1A). Flow cytometry analysis demonstrated that the expression of surface markers conforms to ISCT criteria for MSCs (Additional file 2: Fig. S2, Additional file 3: Fig. S3). *MFN2* knockdown and overexpression efficiency in iPSC-MSCs were confirmed by qRT-PCR and Western blot (Fig. 1B–E). Furthermore, transmission electron microscopy showed that *MFN2* knockdown increased the proportion of spherical mitochondria in iPSC-MSCs, indicating that mitochondrial fusion decreased, and *MFN2* overexpressing increased the length of mitochondria (Additional file 4: Fig. S4A). *MFN2* has no significant effect on the cell proliferation and cell cycle of iPSC-MSCs (Additional file 4: Fig. S4B–D). After 14-day culture in the osteogenic induction medium (IM), *MFN2*-KD iPSC-MSCs showed enhanced mRNA and protein expression of osteogenic differentiation markers RUNX2, OSX, ALP, and OCN (Fig. 1F, H). Alizarin red staining showed more mineralized nodules in the *MFN2*-KD group (Fig. 1I). *MFN2*-OE iPSC-MSCs showed the opposite phenotype (Fig. 1G–I). There is no obvious difference in adipogenic and chondrogenic differentiation ability between the three groups (Additional file 5: Fig. S5). In conclusion, *MFN2* knockdown promotes osteogenic differentiation of iPSC-MSCs.

### Bioinformatics analysis of *MFN2*-KD compared to WT iPSC-MSCs

**Fig. 2:**
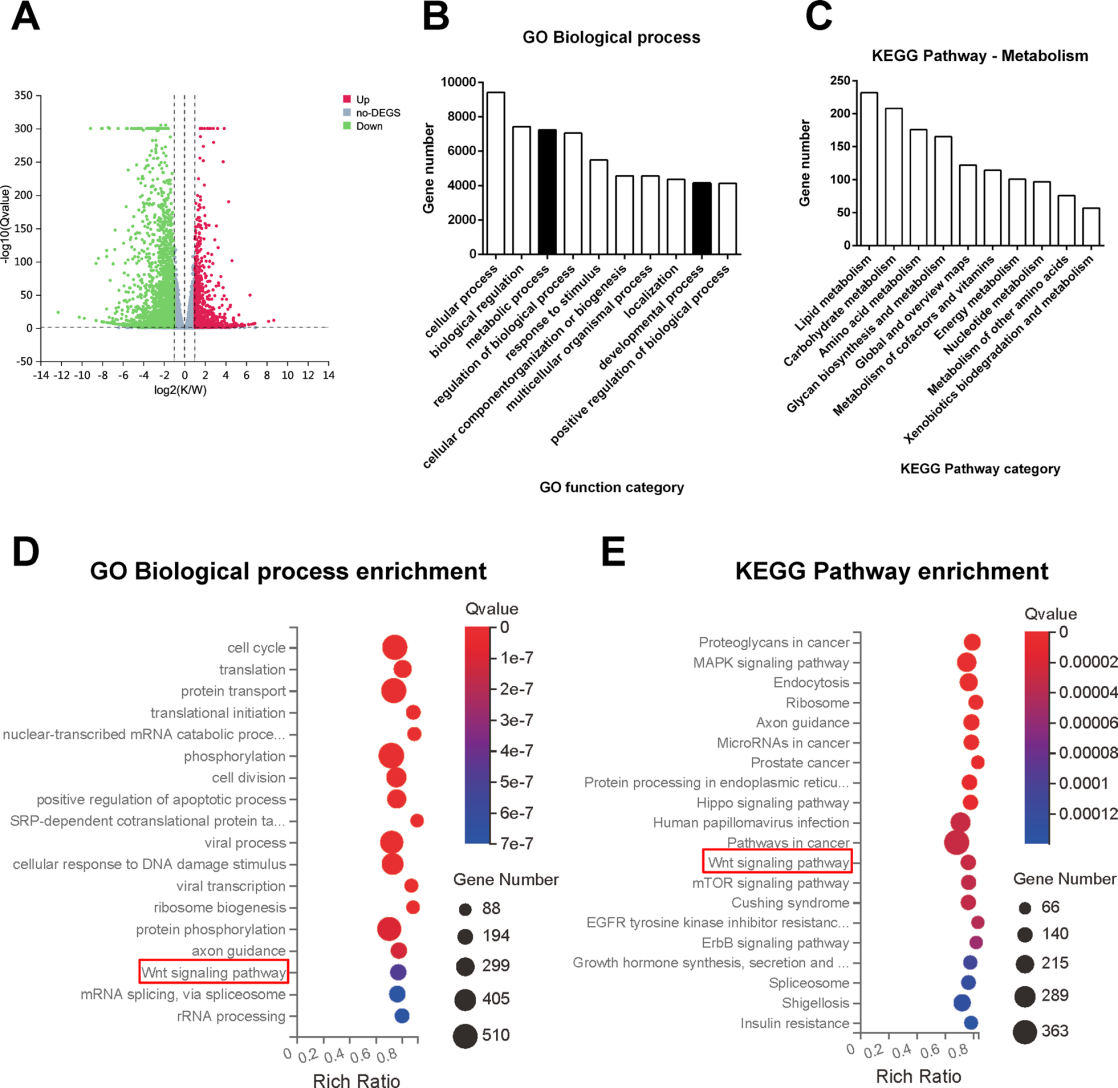
Bioinformatics analysis of *MFN2*-KD compared to WT iPSC-MSCs. **A** The volcano map of differentially expressed genes. Red represents upregulated genes, green represents downregulated genes. **B** Biological process category enrichment histogram of GO analysis. The *X*-axis represents GO biological process terms. **C** Metabolism category enrichment diagram of KEGG pathway analysis. The X-axis represents metabolism terms of KEGG pathway. **D** Biological process category enrichment bubble chart of GO analysis. **E** KEGG pathway enrichment analysis bubble chart. The *Y*-axis of the bubble chart represents GO or KEGG pathway terms, and the color of the bubble represents the Q value of the terms

RNA-sequencing (RNA-seq) was performed to obtain the differential gene expression profile of WT and *MFN2*-KD iPSC-MSCs. There were 5629 upregulated and 5463 downregulated genes in *MFN2*-KD iPSC-MSCs compared with the WT iPSC-MSCs (Fig. 2A), which showed that *MFN2* conferred considerable changes in the transcriptome of the iPSC-MSCs. GO enrichment analysis showed that metabolic and developmental processes were enriched in the biological process (Fig. 2B). The KEGG pathway analysis demonstrated the gene number enriched in the metabolism category (Fig. 2C). This indicated that *MFN2* may play an important role in the differentiation of iPSC-MSCs by regulating cell energy metabolism. Both analyses showed that the Wnt signaling pathway was enriched (Fig. 2D, E). Studies have shown that the ablation of *MFN2* promotes the proliferation and migration of bladder cancer cells by activating the Wnt/β-catenin signaling pathway [16]. *MFN2* promotes the NSC differentiation of hiPSCs by activating the Wnt/β-catenin signaling pathway [18]. Recent studies have shown that depletion of MFN2 in endothelial cells leads to inhibition of β-catenin sulfenylation, thereby activating the activity of the Wnt/β-catenin signaling pathway [19]. Therefore, we focused on the Wnt/β-catenin signaling pathway in the next section.

### *MFN2* knockdown enhances glycolysis of iPSC-MSCs

**Fig. 3:**
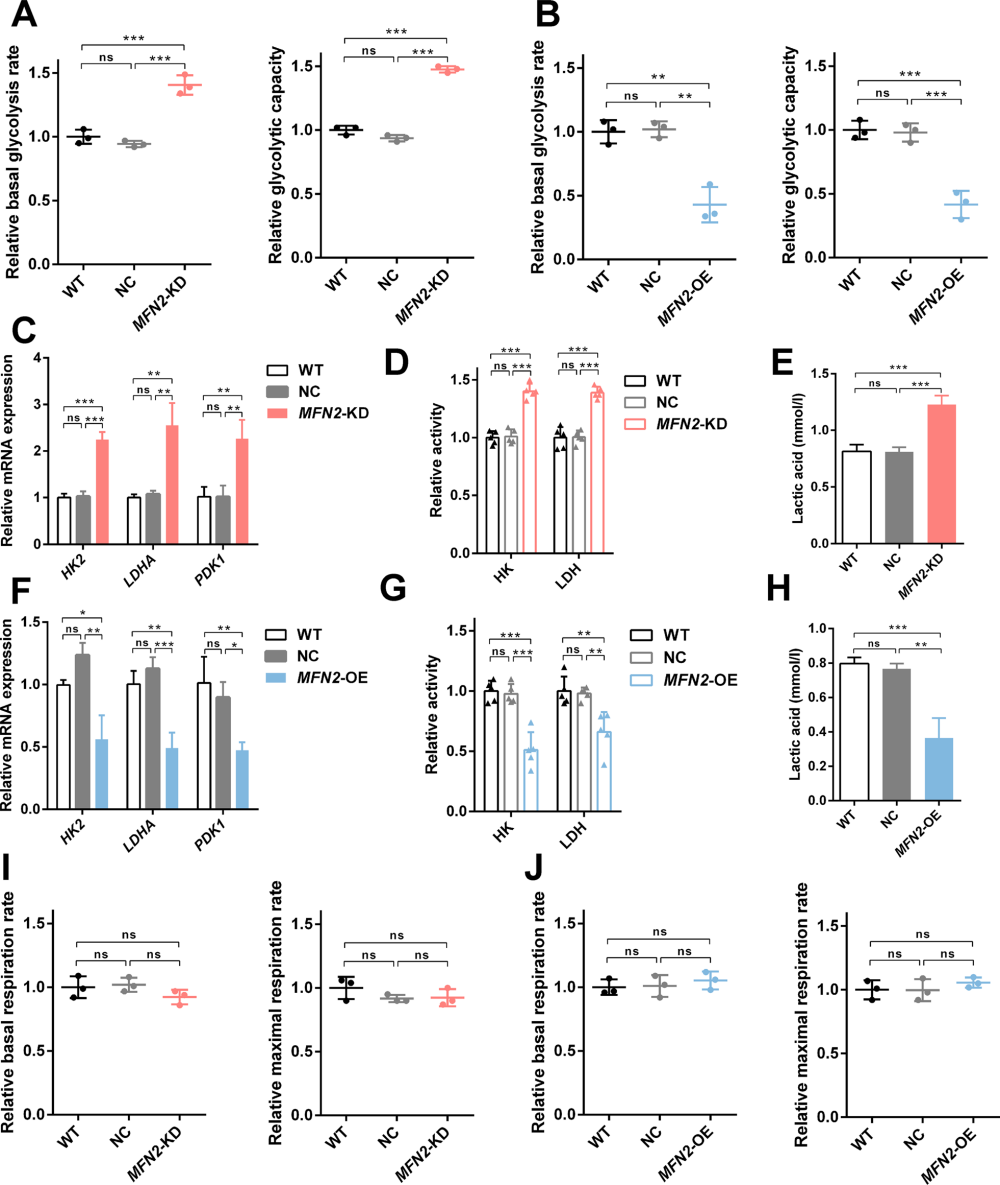
*MFN2* knockdown enhances glycolysis of iPSC-MSCs. **A**–**B** The relative basal glycolysis and glycolytic capacity of WT, *MFN2*-KD, and *MFN2*-OE iPSC-MSCs. **C**, **F** qRT-PCR analysis of glycolytic enzymes in WT, *MFN2*-KD, and *MFN2*-OE iPSC-MSCs. **D**, **G** Relative activity of glycolytic enzymes in WT, *MFN2*-KD, and *MFN2*-OE iPSC-MSCs. **E**, **H** Lactic acid levels of WT, *MFN2*-KD, and *MFN2*-OE iPSC-MSCs. **I**–**J** The relative basal and maximal respiration of WT, *MFN2*-KD, and *MFN2*-OE iPSC-MSCs. NC: negative control. ns, no significant. Data are expressed as mean ± SD, *P < 0.05, **P < 0.01, ***P < 0.001

Glycolysis Stress Test Complete Assay Kit and Mitochondrial Stress Test Complete Assay Kit were utilized to determine whether *MFN2* regulates the energy metabolism of iPSC-MSCs. The results showed that the basal glycolysis and glycolytic capacity in *MFN2*-KD iPSC-MSCs were significantly enhanced (Fig. 3A). We further determined the activity of key enzymes and mRNA expression levels in the glycolysis process and found that they were all significantly upregulated (Fig. 3C, D). Consistently, the concentration of intracellular lactic acid was increased (Fig. 3E). HIF-1α plays an important role in guiding the conversion of cell metabolism to glycolysis, which is required for increased glycolysis by *MFN1/2* ablation [11]. Our result was consistent with it, and *MFN2* knockdown induced the stabilization of HIF-1α (Additional file 6: Fig. S6A, B). There is no statistical difference in the effect of *MFN2* on OXPHOS (Fig. 3I, J). In addition, *MFN2*-OE iPSC-MSCs showed the opposite phenotype (Fig. 3B, F, G, H). These results indicate that *MFN2* knockdown enhances the glycolysis of iPSC-MSCs.

### Wnt/β-catenin signaling pathway regulates glycolysis of iPSC-MSCs

**Fig. 4:**
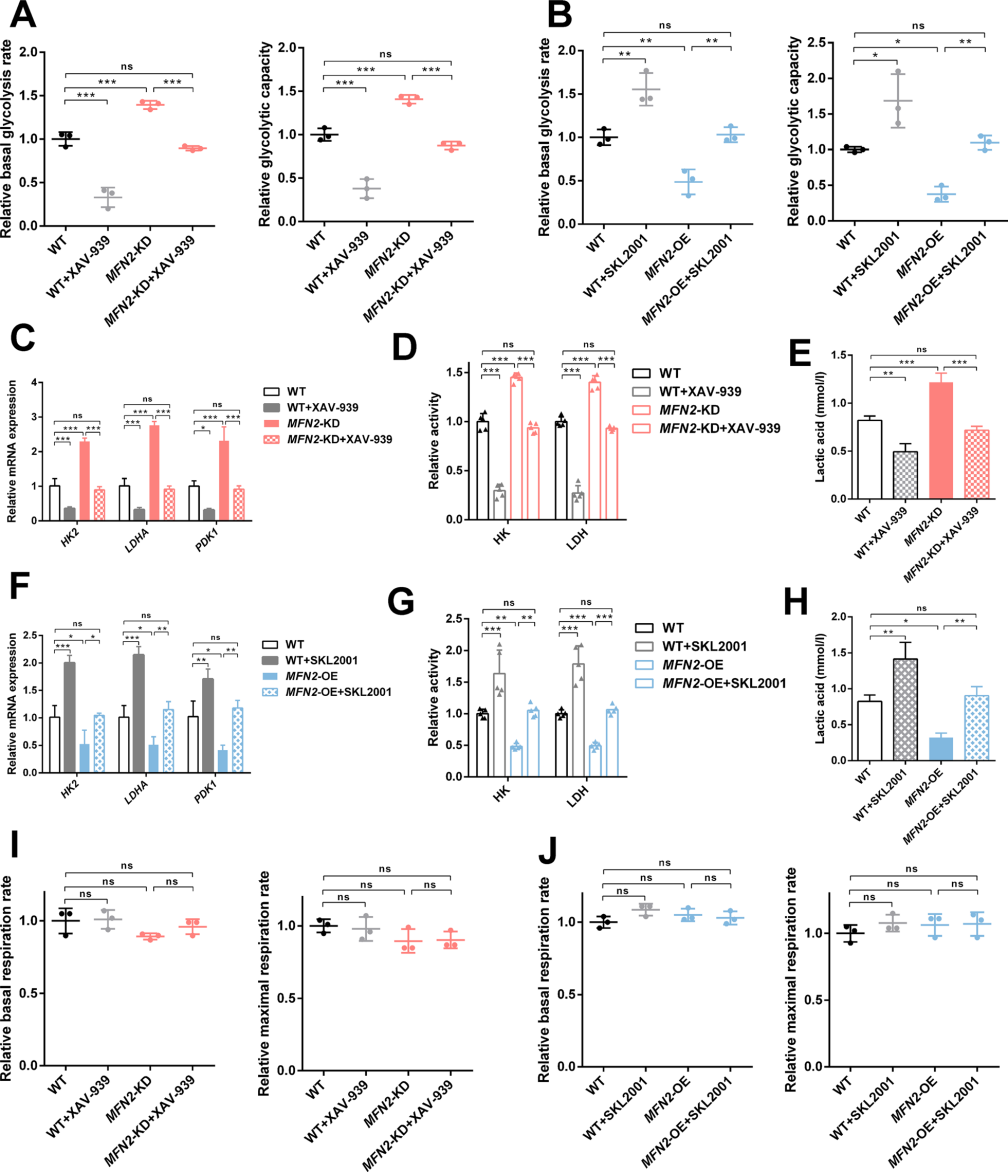
Wnt/β-catenin signaling pathway regulates glycolysis of iPSC-MSCs. **A**–**B** The relative basal glycolysis and glycolytic capacity of WT, *MFN2*-KD, *MFN2*-OE iPSC-MSCs, and cells treated with XAV-939 and SKL2001. **C**, **F** qRT-PCR analysis of glycolytic enzymes in WT, *MFN2*-KD, *MFN2*-OE iPSC-MSCs, and cells treated with XAV-939 and SKL2001. **D**, **G** Relative activity of glycolytic enzymes in WT, *MFN2*-KD, *MFN2*-OE iPSC-MSCs, and cells treated with XAV-939 and SKL2001. **E**, **H** Lactic acid levels of WT, *MFN2*-KD, *MFN2*-OE iPSC-MSCs, and cells treated with XAV-939 and SKL2001. **I**–**J** The relative basal and maximal respiration of WT, *MFN2*-KD, *MFN2*-OE iPSC-MSCs, and cells treated with XAV-939 and SKL2001. ns, no significant. Data are expressed as mean ± SD, *P < 0.05, **P < 0.01, ***P < 0.001

We further determined whether the Wnt/β-catenin signaling pathway was involved in regulating the glycolysis of iPSC-MSCs. XAV-939 (2 μM, Selleck, USA) was used to selectively inhibit Wnt/β-catenin-mediated transcription and SKL2001 (20 μM, Selleck, USA) to disrupt the Axin/β-catenin interaction to activate the Wnt/β-catenin signaling pathway. The results showed that XAV-939 normalized the enhanced glycolysis level *MFN2*-KD iPSC-MSCs, the concentration of intracellular lactic acid, the activity, and mRNA expression level of key glycolysis enzymes (Fig. 4A, C, D, E). SKL2001 rescued the corresponding indicators of *MFN2*-OE iPSC-MSCs (Fig. 4B, F, G, H). Furthermore, inhibitors and activators of the Wnt/β-catenin signaling pathway had no significant effect on OXPHOS (Fig. 4I, J). In conclusion, the Wnt/β-catenin signaling pathway positively regulated the glycolysis of iPSC-MSCs.

### *MFN2* knockdown promotes osteogenic differentiation through aerobic glycolysis via the Wnt/β-catenin signaling pathway

**Fig. 5:**
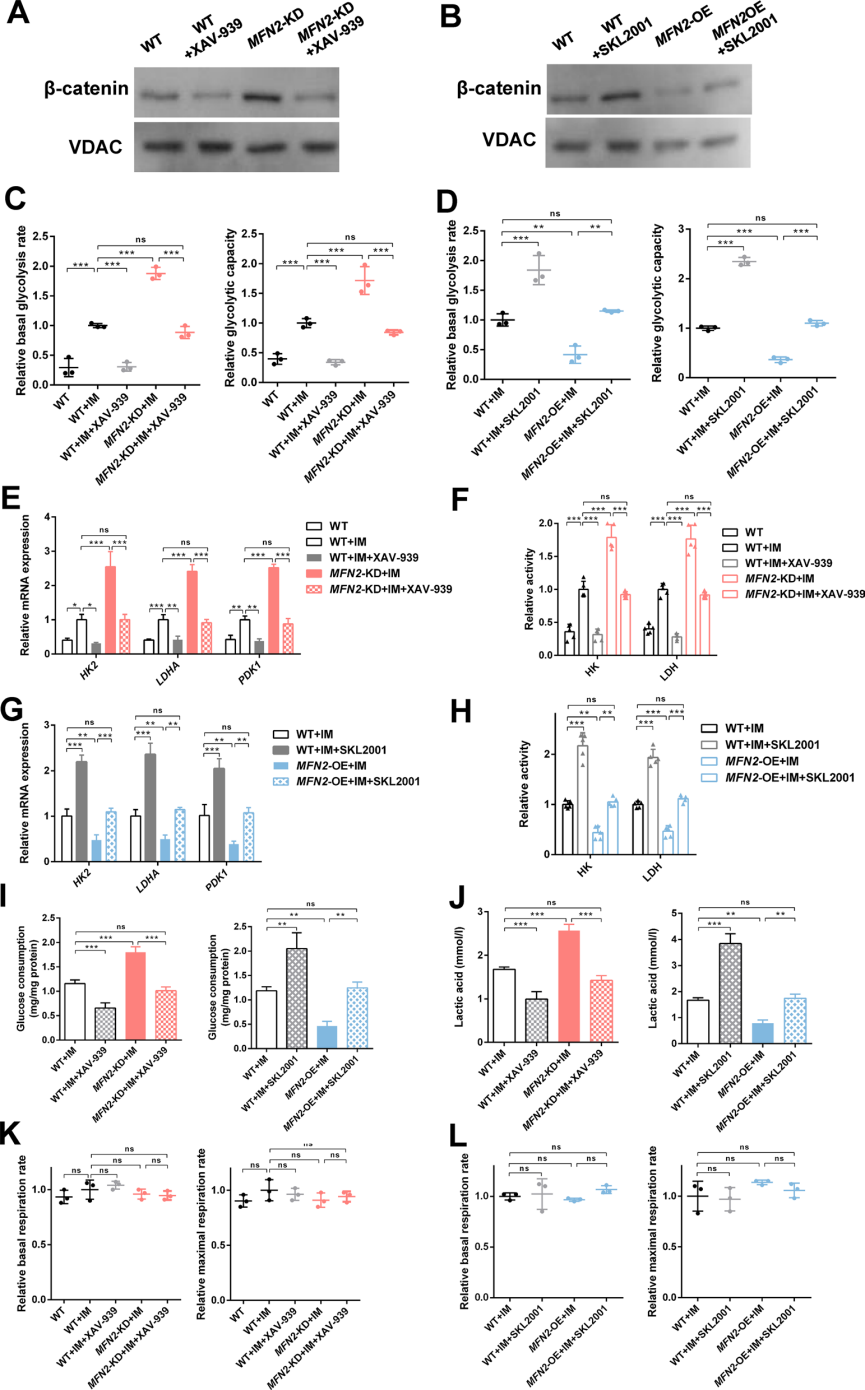
*MFN2* knockdown promotes aerobic glycolysis via the Wnt/β-catenin signaling pathway. **A**, **B** The Western blot analysis of β-catenin in WT, *MFN2*-KD, *MFN2*-OE iPSC-MSCs, and cells treated with XAV-939 and SKL2001 after 24 h of osteogenic induction. **C**–**D** The relative basal glycolysis and glycolytic capacity of WT, *MFN2*-KD, *MFN2*-OE iPSC-MSCs, and cells treated with XAV-939 and SKL2001 after 14 days of osteogenic differentiation. **E**, **G** qRT-PCR analysis of glycolytic enzymes in WT, *MFN2*-KD, *MFN2*-OE iPSC-MSCs, and cells treated with XAV-939 and SKL2001 after 14 days of osteogenic differentiation. **F**, **H** Relative activity of glycolytic enzymes in WT, *MFN2*-KD, *MFN2*-OE iPSC-MSCs, and cells treated with XAV-939 and SKL2001 after 14 days of osteogenic differentiation. **I** The glucose consumption rate of WT, *MFN2*-KD, *MFN2*-OE iPSC-MSCs, and cells treated with XAV-939 and SKL2001 after 14 days of osteogenic differentiation. **J** Lactic acid levels of WT, *MFN2*-KD, *MFN2*-OE iPSC-MSCs, and cells treated with XAV-939 and SKL2001 after 14 days of osteogenic differentiation. **K-L** The relative basal and maximal respiration of WT, *MFN2*-KD, *MFN2*-OE iPSC-MSCs, and cells treated with XAV-939 and SKL2001 after 14 days of osteogenic differentiation. IM, osteogenic induction medium. ns, no significant. Data are expressed as mean ± SD, *P < 0.05, **P < 0.01, ***P < 0.001

To further determine the role of *MFN2* and the Wnt/β-catenin signaling pathway in the osteogenic differentiation and energy metabolism of iPSC-MSCs, we first determine the effect of *MFN2* on the Wnt/β-catenin signaling pathway. Immunoprecipitation showed a binding relationship between *MFN2* and β-catenin (Additional file 7: Fig. S7). After osteogenic induction with/without inhibitor/activator treatment for 24 h, Western blot results showed that *MFN2* knockdown increased the expression level of β-catenin in iPSC-MSCs, which was normalized by XAV-939 (Fig. 5A). The expression level of β-catenin in *MFN2*-OE iPSC-MSCs decreased, which was rescued by SKL2001 (Fig. 5B). Therefore, *MFN2* regulates the Wnt/β-catenin signaling pathway by directly binding to β-catenin.

We next determined the effects of *MFN2* and Wnt/β-catenin signaling pathways on energy metabolism in the osteogenic differentiation of iPSC-MSCs. The results showed that *MFN2* knockdown significantly increased glucose consumption, glycolysis level, intracellular lactic acid concentration, activity, and mRNA expression levels of key glycolysis enzymes and HIF-1α 14 days after osteogenic differentiation, while XAV-939 eliminated this effect (Fig. 5C, E, F, I, J and Additional file 6: Fig. S6C, D). SKL2001 rescued the reduced glycolysis level of *MFN2*-OE iPSC-MSCs (Fig. 5D, G, H, I, J and Additional file 6: Fig. S6E, F). XAV-939 and SKL2001 had no significant effect on OXPHOS during osteogenic differentiation (Fig. 5K, L).

**Fig. 6:**
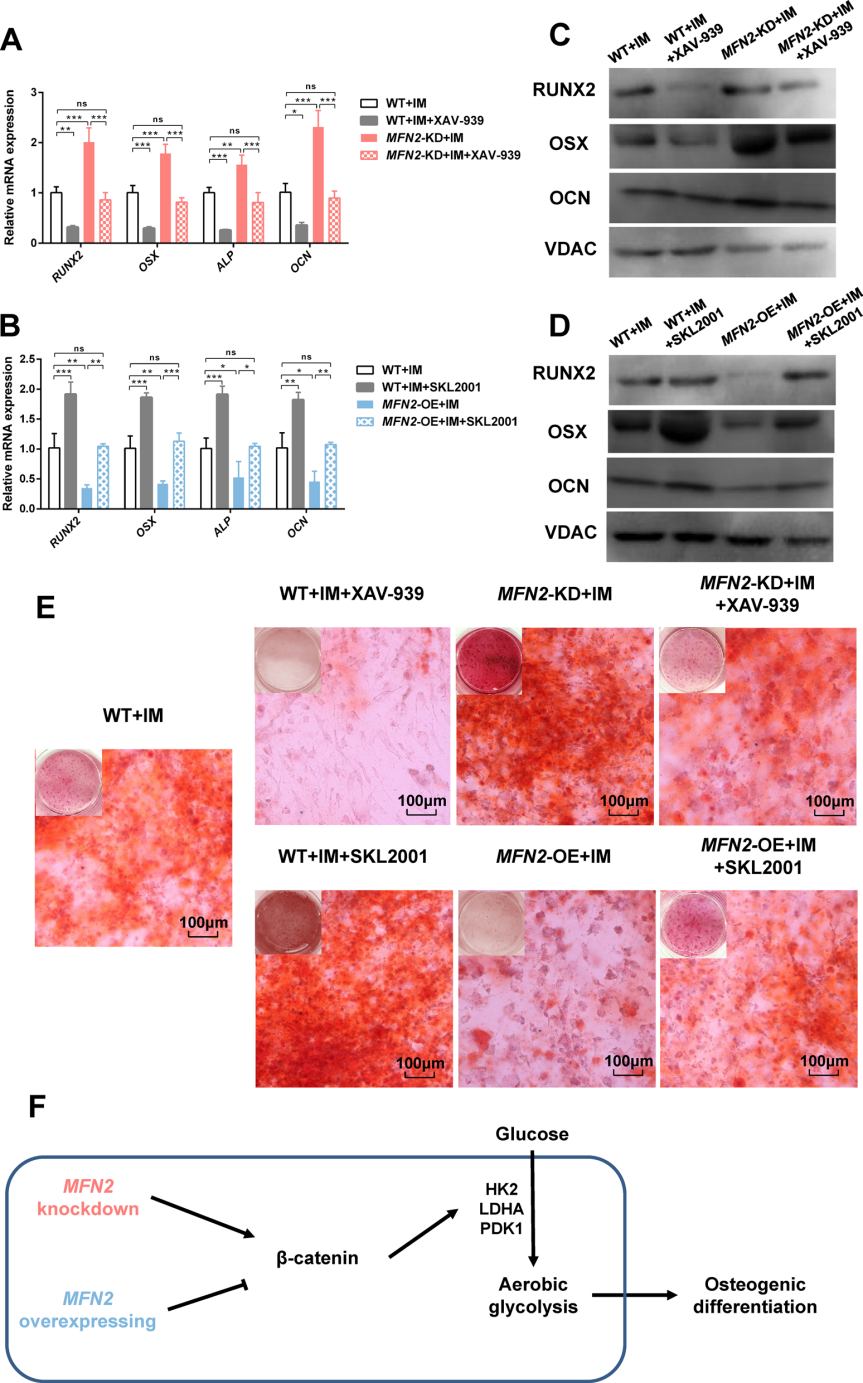
*MFN2* knockdown promotes osteogenic differentiation through aerobic glycolysis via the Wnt/β-catenin signaling pathway. **A**–**B** qRT-PCR analysis of osteogenic differentiation markers in WT, *MFN2*-KD, *MFN2*-OE iPSC-MSCs, and cells treated with XAV-939 and SKL2001 after 14 days of osteogenic differentiation. **C**–**D** Western blot analysis of osteogenic differentiation markers in WT, *MFN2*-KD, *MFN2*-OE iPSC-MSCs, and cells treated with XAV-939 and SKL2001 after 14 days of osteogenic differentiation. **E** Alizarin red staining of WT, *MFN2*-KD, *MFN2*-OE iPSC-MSCs, and cells treated with XAV-939 and SKL2001 after 14 days of osteogenic differentiation. **F** The interaction between *MFN2*, β-catenin, aerobic glycolysis, and osteogenic differentiation. IM, osteogenic induction medium. ns, no significant. Data are expressed as mean ± SD, *P < 0.05, **P < 0.01, ***P < 0.001

**Fig. 7:**
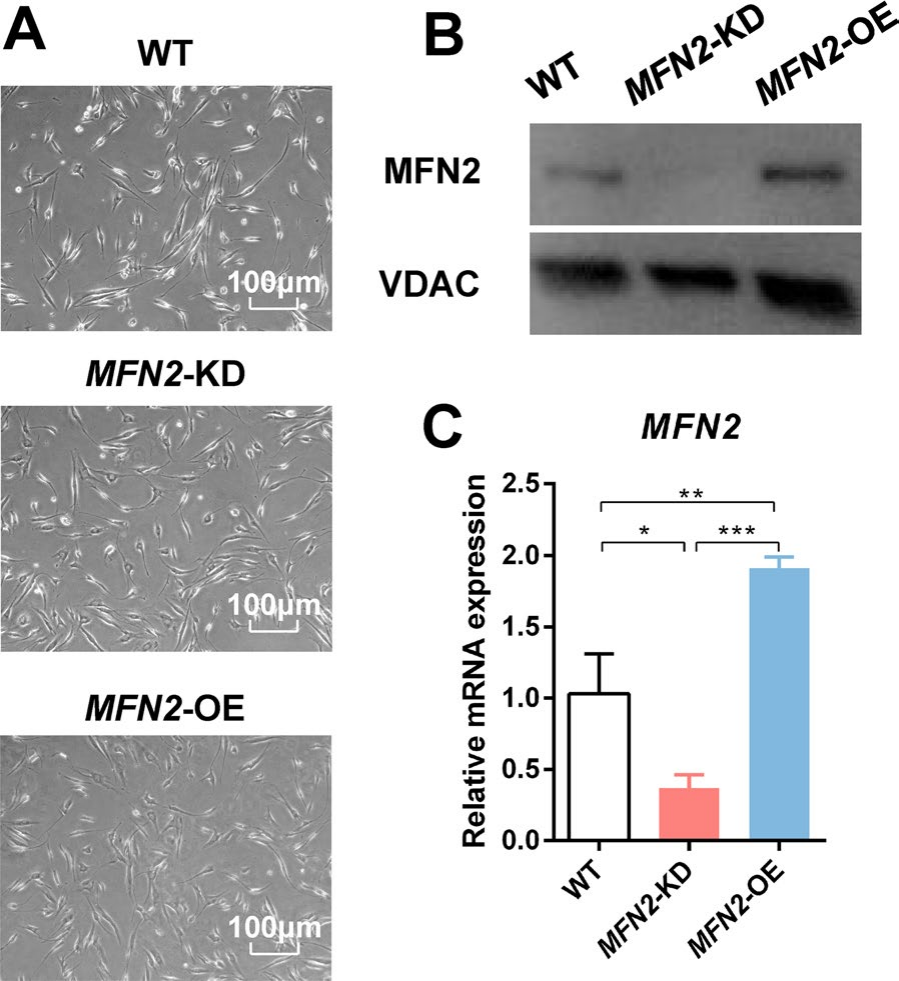
Construction of *MFN2*-KD and *MFN2*-OE PDLSCs. **A** The morphology of WT, *MFN2*-KD, and *MFN2*-OE PDLSCs. **B**–**C** Western blot and qRT-PCR analysis of MFN2 in WT, *MFN2*-KD, and *MFN2*-OE PDLSCs.  *P < 0.05, **P < 0.01, ***P < 0.001

**Fig. 8:**
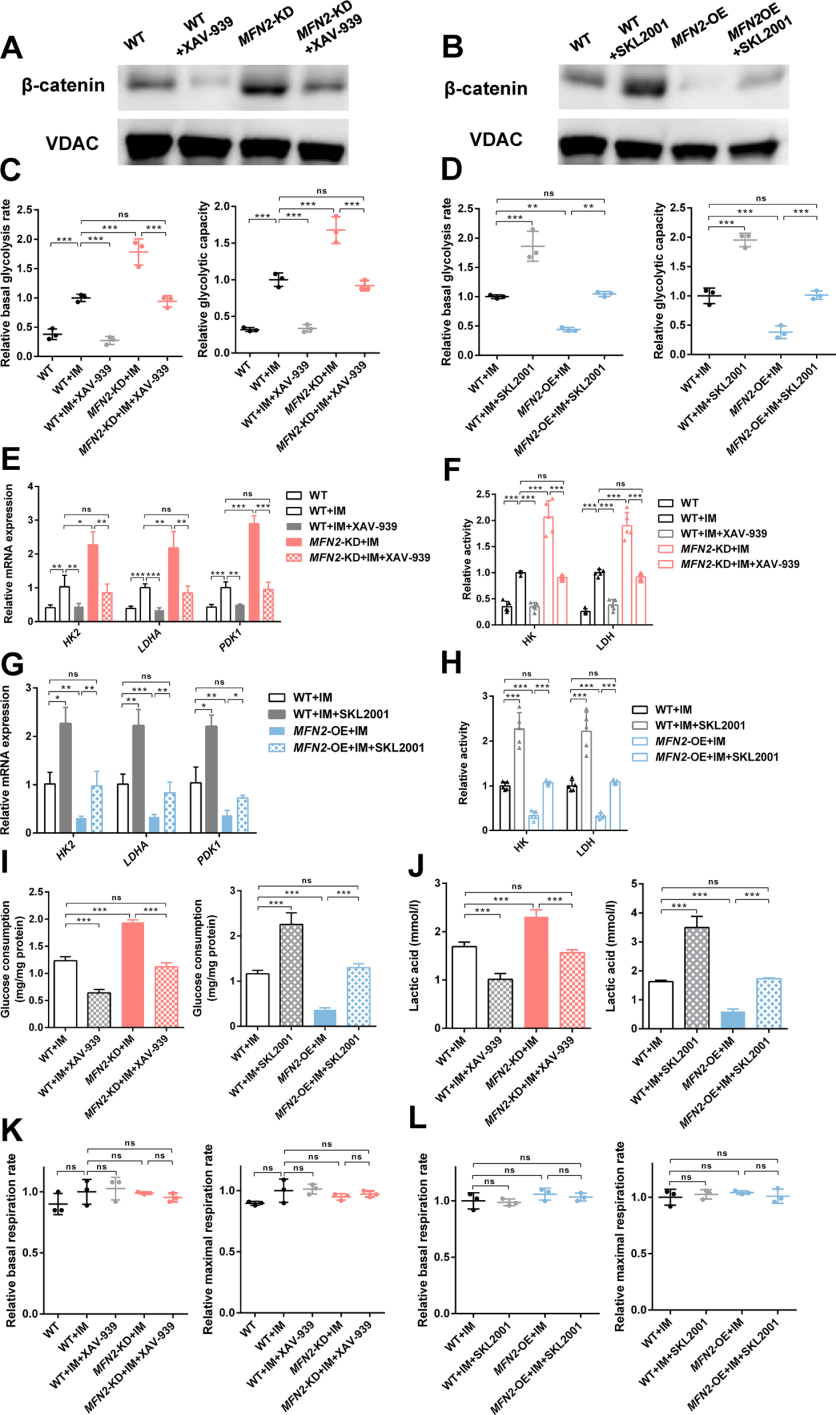
*MFN2* knockdown promotes aerobic glycolysis via the Wnt/β-catenin signaling pathway in PDLSCs. **A**–**B** The Western blot analysis of β-catenin in WT, *MFN2*-KD, *MFN2*-OE PDLSCs, and cells treated with XAV-939 and SKL2001 after 24 h of osteogenic induction. **C**–**D** The relative basal glycolysis and glycolytic capacity of WT, *MFN2*-KD, *MFN2*-OE PDLSCs, and cells treated with XAV-939 and SKL2001 after osteogenic differentiation. **E**, **G** qRT-PCR analysis of glycolytic enzymes in WT, *MFN2*-KD, *MFN2*-OE PDLSCs, and cells treated with XAV-939 and SKL2001 after osteogenic differentiation. **F**, **H** Relative activity of glycolytic enzymes in WT, *MFN2*-KD, *MFN2*-OE PDLSCs, and cells treated with XAV-939 and SKL2001 after osteogenic differentiation. **I** The glucose consumption rate of WT, *MFN2*-KD, *MFN2*-OE PDLSCs, and cells treated with XAV-939 and SKL2001 after osteogenic differentiation. **J** Lactic acid levels of WT, *MFN2*-KD, *MFN2*-OE PDLSCs, and cells treated with XAV-939 and SKL2001 after osteogenic differentiation. **K**–**L** The relative basal and maximal respiration of WT, *MFN2*-KD, *MFN2*-OE PDLSCs, and cells treated with XAV-939 and SKL2001 after osteogenic differentiation. IM, osteogenic induction medium. ns, no significant. Data are expressed as mean ± SD, *P < 0.05, **P < 0.01, ***P < 0.001

**Fig. 9:**
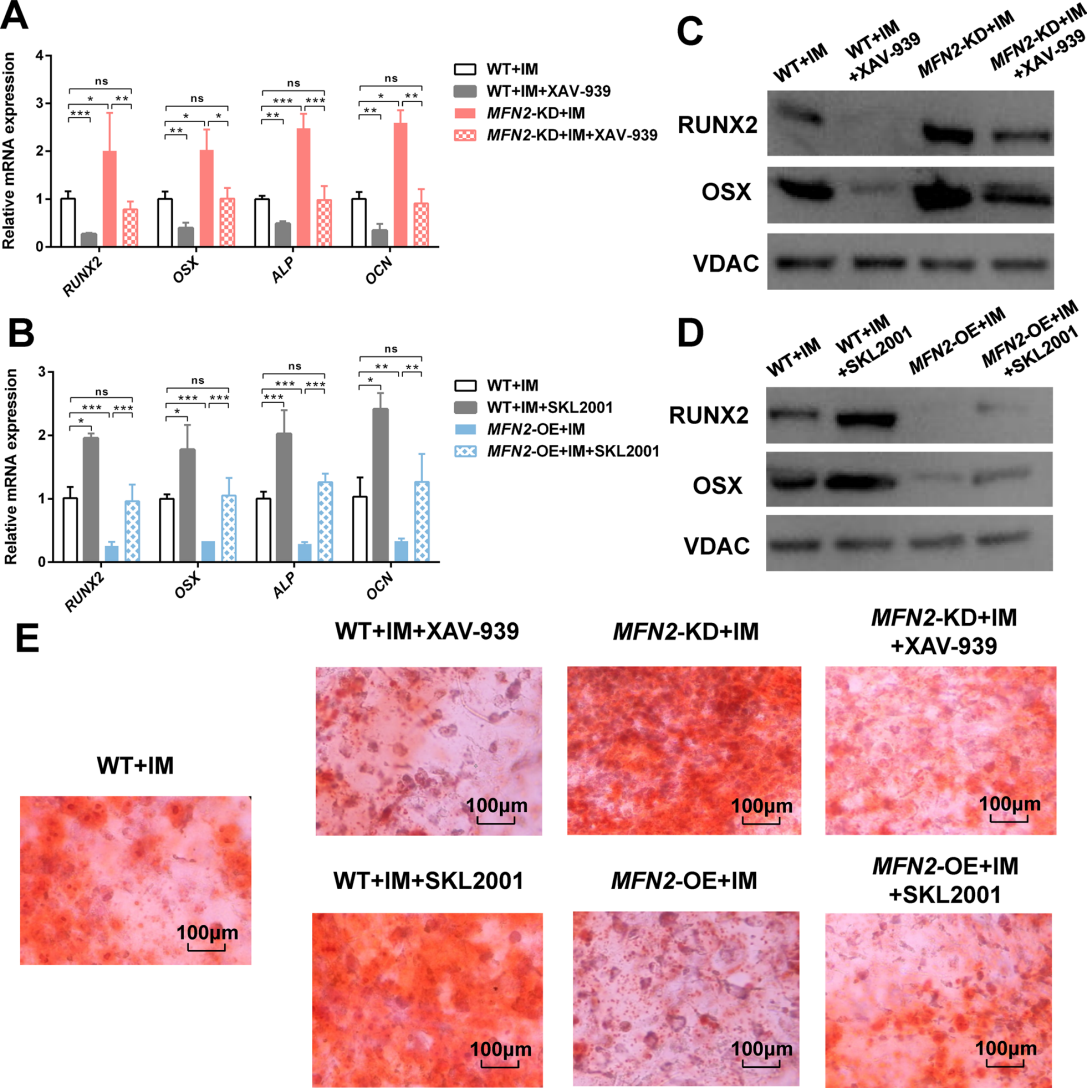
*MFN2* knockdown promotes osteogenic differentiation through aerobic glycolysis via the Wnt/β-catenin signaling pathway in PDLSCs. **A**–**B** qRT-PCR analysis of osteogenic differentiation markers in WT, *MFN2*-KD, *MFN2*-OE PDLSCs, and cells treated with XAV-939 and SKL2001 after osteogenic differentiation. **C**–**D** Western blot analysis of osteogenic differentiation markers in WT, *MFN2*-KD, *MFN2*-OE PDLSCs, and cells treated with XAV-939 and SKL2001 after osteogenic differentiation. **E** Alizarin red staining of WT, *MFN2*-KD, *MFN2*-OE PDLSCs, and cells treated with XAV-939 and SKL2001 after osteogenic differentiation. IM, osteogenic induction medium. ns, no significant. Data are expressed as mean ± SD, *P < 0.05, **P < 0.01, ***P < 0.001

Finally, we evaluated the effects of *MFN2* and Wnt/β-catenin signaling pathways on osteogenic differentiation. Consistent with changes in energy metabolism, we found that the increased expression of osteogenic differentiation markers (Fig. 6A, C) and mineralized nodules of *MFN2*-KD iPSC-MSCs (Fig. 6E) were normalized by XAV-939. The decreased osteogenic differentiation of *MFN2*-OE iPSC-MSCs was partially rescued by SKL2001 (Fig. 6B, D, E). We further used PDLSCs as a cell model and got consistent results as iPSC-MSCs (Figs. 7, 8 and 9). These results indicate that downregulated *MFN2* promotes osteogenic differentiation of MSCs through aerobic glycolysis via the Wnt/β-catenin signaling pathway (Fig. 6F).

## Discussion

MSCs are progenitor cells that can differentiate into osteoblasts, adipocytes, and chondrocytes [20]. Osteoblasts synthesize numerous extracellular matrix proteins and therefore have a high demand for materials and energy sources during the osteogenic differentiation of MSCs. There is controversy about the metabolic mode of MSCs’ osteogenic differentiation. Recent studies have found that the OXPHOS is activated during the osteogenic differentiation of MSCs, as revealed by the upregulation of the oxygen consumption rate of mitochondria and respiratory enzymes, while glycolysis is reduced [21, 22]. Other studies found that ATP production of hMSCs is more dependent on glycolysis compared with differentiated cells [23]. Inhibition of glycolytic enzymes in vitro impairs bone formation in mouse osteoblasts [24]. The activation of glycolysis by overexpression of HIF-1α leads to more osteoblasts and bone formation in mice [25]. This raises the question of why aerobic glycolysis, which produces less ATP, is chosen in osteoblasts. One possible explanation is that osteoblasts have important requirements that exceed ATP, aerobic glycolysis may be necessary to meet the specific metabolic needs of osteogenic differentiation. Another possible explanation is that the cell culture environment has sufficient energy sources, so inefficient ATP production is not a problem [3]. Studies have shown that osteogenic differentiation depends on changes in specific stages of energy acquisition while early osteogenic differentiation requires the synthesis of collagen and upregulation of OXPHOS. The increase in OXPHOS activity is accompanied by the increase in reactive oxygen species (ROS), which may lead to cell damage or lineage switching. Therefore, cell metabolism is converted to glycolysis during the maturation and mineralization of osteoblasts [26, 27]. Our conclusion provides a compromise: Aerobic glycolysis is activated during iPSC-MSCs osteogenesis, and the OXPHOS level is unchanged. This indicates the metabolic plasticity of the osteogenic differentiation of MSCs in meeting cellular energy demands.

Mitochondria are important organelles for cells to synthesize ATP and critical for maintaining normal physiological functions. Mitochondria change their shape via GTPases (mitofusin-1 (MFN1), MFN2, optic atrophy-1 (OPA1), fission protein -1 (FIS1), and dynamin-related protein-1 (DRP1)) to respond to changes in cell energy demand and supply [28]. There are few studies on *MFN2* and osteogenic differentiation. The lack of *Mfn2* in the mouse osteoblast lineage enhances the formation of basal bone in vivo and in vitro by increasing the number of osteoblasts and osteogenic differentiation capability [12]. The expression of *MFN2* and the endoplasmic reticulum-mitochondrial coupling increase in the inflammatory microenvironment, leading to impaired osteogenic differentiation of periodontal ligament stem cells [29]. This is consistent with our results. However, some studies have shown that *Mfn2* increases during the differentiation of mouse osteoblasts, and *Mfn2* knockdown reduces mitochondrial elongation and osteogenic differentiation [30]. Conflicting results may be due to differences in donor species, cell type, induction medium, and oxygen concentration. The depletion of *MFN2* in different cell models changes the cellular metabolic profile, resulting in a decrease in cell oxygen consumption and OXPHOS level. The cells increase glucose uptake and turn to glycolysis to compensate for the decreased ATP production [7]. Our glycolysis results are consistent with it. But interestingly, our results showed that there is no significant difference in the effect of *MFN2* on OXPHOS. Recent studies have shown that MFN1 regulates the metastasis of hepatocellular carcinoma through a metabolic shift from aerobic glycolysis to OXPHOS [31]. Knockdown of the other two mitochondrial fusion regulatory genes *OPA1* or *MFN1* attenuates OXPHOS and ATP production of tumor cells [32]. We speculate that there may be a certain compensatory effect between different mitochondrial fusion genes and the level of cellular OXPHOS, thereby preventing the aerobic metabolism and ATP production of cells from experiencing drastic fluctuations. Studies have shown that *MFN2* plays an important role in regulating cell proliferation [7]. Interestingly, our study demonstrated that *MFN2* has no significant effect on the cell proliferation and cell cycle of iPSC-MSCs, which may be the result of different cell types or mechanisms [33]. Therefore, *MFN2* knockdown does not stimulate osteogenic differentiation by regulating cell proliferation.

In addition, we want to figure out the signaling pathway that mediates glycolysis. Although most signal pathways related to development have been shown to regulate osteogenic differentiation, their potential effects on cell metabolism have not been fully studied. The Wnt signaling pathway is critical in differentiation and development, which plays an important role in normal bone development and homeostasis [34]. Recent studies have shown that activation of the canonical Wnt/β-catenin signaling pathway rescues glycolytic and osteogenic differentiation disorders [35]. Wnt signaling pathway reprograms cell glucose metabolism during osteogenic differentiation. WNT3A induces aerobic glycolysis by increasing the levels of key glycolytic enzymes, thereby enhancing bone formation in vitro and in vivo [24]. We found that blocking the Wnt/β-catenin signaling pathway normalized the enhanced glycolysis and mineralization of *MFN2*-KD iPSC-MSCs, while the activation rescued the defects in *MFN2*-OE iPSC-MSCs. Interestingly, activating or inhibiting the Wnt/β-catenin signaling pathway has almost no significant effect on OXPHOS in all cells. However, a recent study shows that Wnt/β-catenin inhibition increases OXPHOS in colorectal cancer cells [36]. We think this may be due to the existence of other pathways coregulating OXPHOS during the osteogenic differentiation of iPSC-MSCs, or the existence of abnormal metabolic regulation mechanisms in cancer cells.

*MFN2* may regulate osteogenic differentiation through multiple signaling pathways. *MFN2* inhibits mTORC2 signaling by binding to its HR1 domain [37]. mTORC2 signal positively regulates bone development and homeostasis [38]. *MFN2* interacts with the Notch signaling pathway [39], which plays an important role in bone development and remodeling [40]. Studies have found that increasing OXPHOS by acetylation stabilizes β-catenin, thereby promoting the osteogenic differentiation of BMSCs, which represents a possible mechanism in which the interaction between *MFN2* and Wnt/β-catenin signaling pathway is directly related to osteogenic differentiation [41]. There are few studies on the direct relationship between *MFN2* and Wnt/β-catenin pathway. Recent studies have shown that MFN2 significantly interacts with β-catenin in endothelial cells. Sequence alignment analysis shows that MFN2 has a β-catenin binding motif between its HR domains. Although MFN2 is mainly located in the cytoplasm and mitochondria, both MFN2 and β-catenin can accumulate in the nucleus. Depletion of MFN2 leads to inhibition of β-catenin sulfenylation, thereby activating the transcriptional activity of β-catenin [19]. Similarly, our results showed that MFN2 can bind to β-catenin. The Wnt/β-catenin signaling pathway regulates the proliferation and migration of bladder cancer, which can be activated by the ablation of *MFN2* [16]. This is consistent with our results. But another recent study found that *MFN2* overexpression promotes the NSC differentiation of hiPSCs by activating the Wnt/β-catenin signaling pathway [18]. This may be due to the interaction between *MFN2* and Wnt/β-catenin signaling pathway may play different roles in different kinds of differentiation. Further work is needed to figure out the sites of action and mechanism between *MFN2* and Wnt/β-catenin signaling pathway.

Our work has some limitations. Firstly, we have not validated our conclusions in vivo, because there are certain differences in the microenvironment in vivo and in vitro, which may lead to differences in results. Secondly, fatty acids and glutamine are utilized as energy sources for cell metabolism in addition to glucose [42]. We only detected the changes in glucose uptake and did not further explore the possible roles and mechanisms of the other two raw materials in the osteogenic differentiation of iPSC-MSCs. Thirdly, MSCs can regulate the innate and adaptive immune system, which immunomodulatory activity is mainly mediated by paracrine factors [43]. Mitochondria are increasingly recognized as critical organelles for innate and adaptive immune signals. *MFN2* is essential for cytokine production, antigen processing, and phagocytosis [44]. Further work needs to be done to get a better understanding of the function of *MFN2* and serve the periodontal tissue regeneration.

## Conclusions

In summary, our study clarified that the lack of *MFN2* promoted the osteogenic differentiation of iPSC-MSCs, and increased glucose consumption and lactic acid production in the presence of sufficient oxygen, as well as the expression of glycolytic enzymes, which promoted aerobic glycolysis. Inhibition of the Wnt/β-catenin signaling pathway normalized the enhanced aerobic glycolysis and osteogenic differentiation of *MFN2*-KD iPSC-MSCs. Overexpression of *MFN2* can lead to the opposite phenotype. This study identified *MFN2* as a new regulator of MSCs’ cell metabolism, which is expected to provide a new theoretical basis and therapeutic target for alveolar bone repair, periodontal regeneration, moreover, prevention and treatment of diseases related to osteogenic differentiation and abnormal bone formation.

## Supplementary Information

**Additional file 1: Fig. S1.** The morphology of iPSCs and immunofluorescence analysis of pluripotency markers.

**Additional file 2: Fig. S2.** Flow cytometry analysis of MSC surface markers. Flow cytometry analysis of CD73, CD90 and CD105 in WT (**A**), *MFN2*-KD (**B**), and *MFN2*-OE iPSC-MSCs (**C**).

**Additional file 3: Fig. S3.** Flow cytometry analysis of hematopoietic cell surface markers. Flow cytometry analysis of CD34 and CD45 in WT (**A**), *MFN2*-KD (**B**), and *MFN2*-OE iPSC-MSCs (**C**).

**Additional file 4: Fig. S4.** Mitochondrial morphology, cell proliferation, and cell cycle analysis of WT, *MFN2*-KD and *MFN2*-OE iPSC-MSCs. **A** Mitochondrial morphology (white arrow) of WT, *MFN2*-KD and *MFN2*-OE iPSC-MSCs under the transmission electron microscope. **B** The CCK-8 assay for WT, *MFN2*-KD and *MFN2*-OE iPSC-MSCs. **C–D** Cell cycle analysis of WT, *MFN2*-KD and *MFN2*-OE iPSC-MSCs. ns, no significant.

**Additional file 5: Fig. S5.** Oil Red O staining and Alcian blue staining of WT, *MFN2*-KD and *MFN2*-OE iPSC-MSCs after adipogenic and chondrogenic differentiation.

**Additional file 6: Fig. S6.** qRT-PCR (**A**) and Western blot analysis (**B**) of HIF-1α in WT, *MFN2*-KD, *MFN2*-OE iPSC-MSCs. qRT-PCR (**C**, **E**) and Western blot analysis (**D**, **F**) of HIF-1α in WT, *MFN2*-KD, *MFN2*-OE iPSC-MSCs, and cells treated with XAV-939 and SKL2001 after osteogenic differentiation.

**Additional file 7: Fig. S7.** Immunoprecipitation assay detected the binding relationship between MFN2 and β-catenin.

## Data Availability

All data generated or analyzed during this study are included in this published article. The RNA-seq data have been uploaded to SRA (SRR16296512, SRR16296513, SRR16296514, SRR16296515, SRR16296516, SRR16296517).

## Abbreviations

*MFN2*: Mitofusin 2
iPSCs: Induced pluripotent stem cells
MSCs: Mesenchymal stem cells
iPSC-MSCs: Induced pluripotent stem cell-derived mesenchymal stem cells
*MFN2*-KD: *MFN2* knockdown
*MFN2*-OE: *MFN2* overexpressing
WT: Wild type
NC: Negative control
OXPHOS: Oxidative phosphorylation
HK2: Hexokinase-2
LDHA: Lactate dehydrogenase A
PDK1: Pyruvate dehydrogenase kinase 1
IM: Osteogenic induction medium
qRT-PCR: Quantitative real-time polymerase chain reaction
RNA-seq: RNA-sequencing
KEGG: Kyoto Encyclopedia of Genes and Genomes
GO: Gene Ontology
SD: Standard deviation
HIF-1α: Hypoxia-inducible factor 1α
