# Supplementary figures and images for: MFN2 knockdown promotes osteogenic differentiation of iPSC-MSCs through aerobic glycolysis mediated by the Wnt/β-catenin signaling pathway
Source: Stem Cell Res Ther. 2022 Apr 12;13:162. doi: 10.1186/s13287-022-02836-w (PMC9006575; doi:10.1186/s13287-022-02836-w)

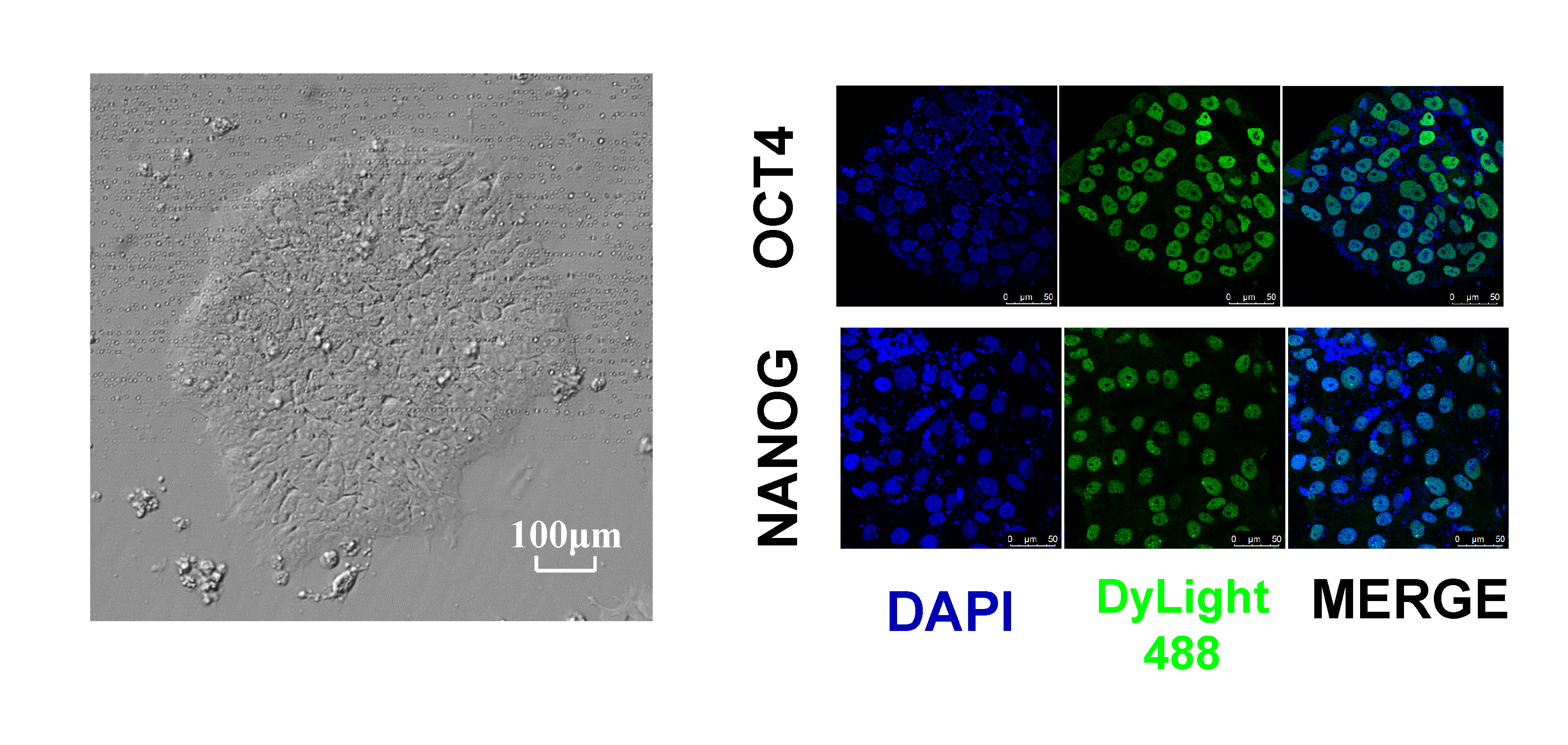

Supplement: Supplementary file 1 — Additional file 1: Fig. S1. The morphology of iPSCs and immunofluorescence analysis of pluripotency markers. [file 13287_2022_2836_MOESM1_ESM.tiff]

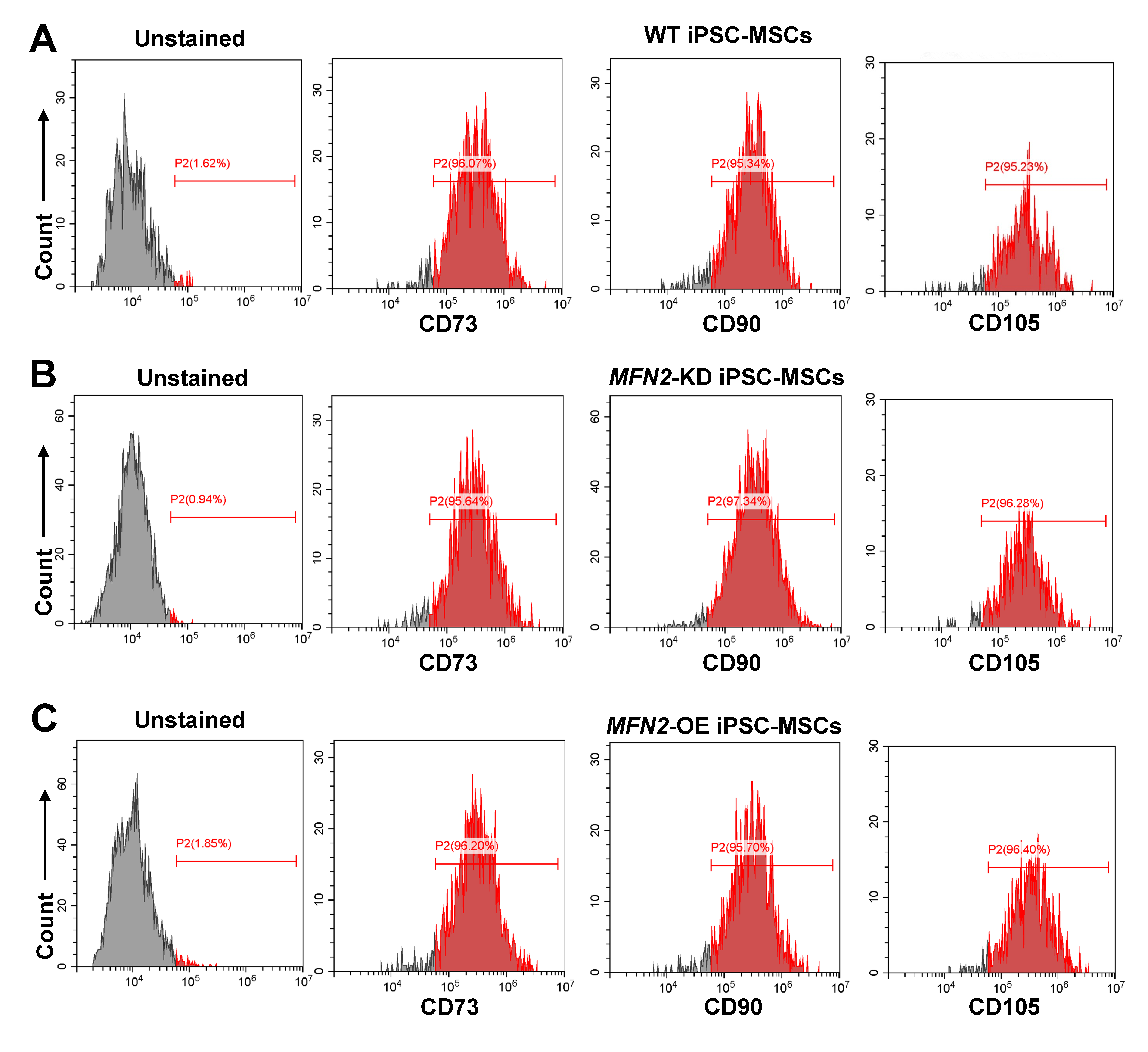

Supplement: Supplementary file 2 — Additional file 2: Fig. S2. Flow cytometry analysis of MSC surface markers. Flow cytometry analysis of CD73, CD90 and CD105 in WT (A), MFN2-KD (B), and MFN2-OE iPSC-MSCs (C). [file 13287_2022_2836_MOESM2_ESM.tiff]

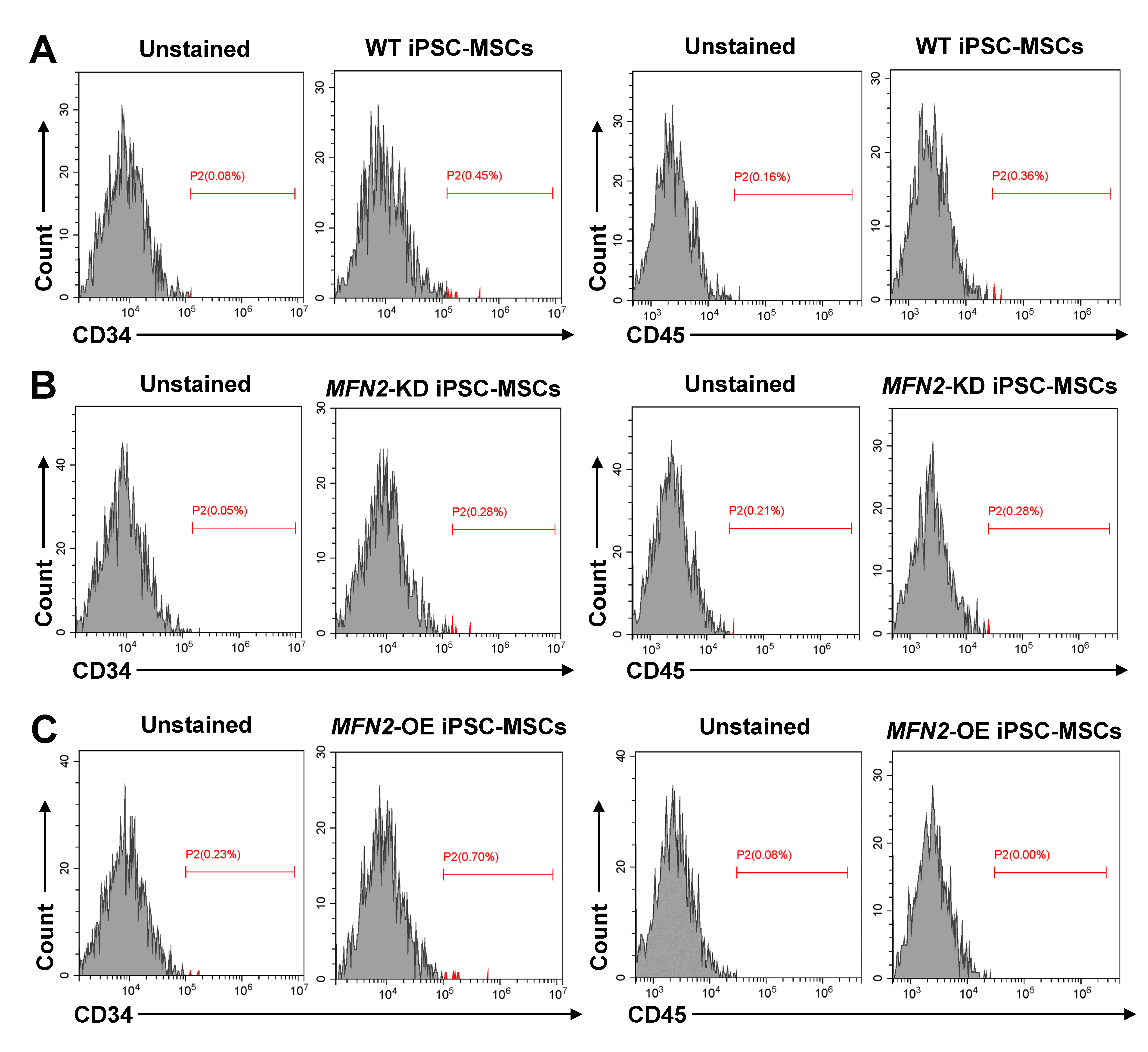

Supplement: Supplementary file 3 — Additional file 3: Fig. S3. Flow cytometry analysis of hematopoietic cell surface markers. Flow cytometry analysis of CD34 and CD45 in WT (A), MFN2-KD (B), and MFN2-OE iPSC-MSCs (C). [file 13287_2022_2836_MOESM3_ESM.tiff]

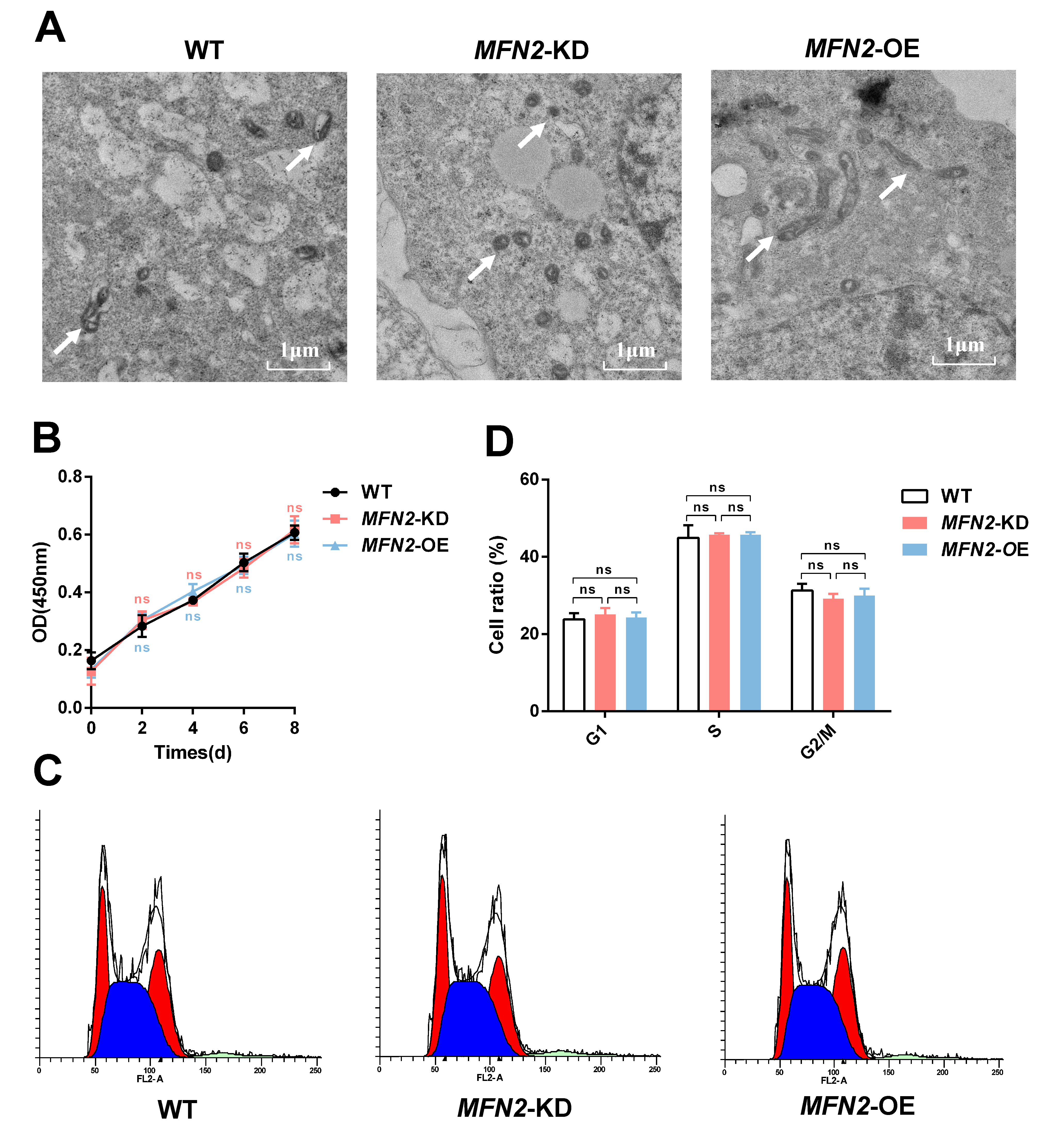

Supplement: Supplementary file 4 — Additional file 4: Fig. S4. Mitochondrial morphology, cell proliferation, and cell cycle analysis of WT, MFN2-KD and MFN2-OE iPSC-MSCs. A Mitochondrial morphology (white arrow) of WT, MFN2-KD and MFN2-OE iPSC-MSCs under the transmission electron microscope. B The CCK-8 assay for WT, MFN2-KD and MFN2-OE iPSC-MSCs. C–D Cell cycle analysis of WT, MFN2-KD and MFN2-OE iPSC-MSCs. ns, no significant. [file 13287_2022_2836_MOESM4_ESM.tif]

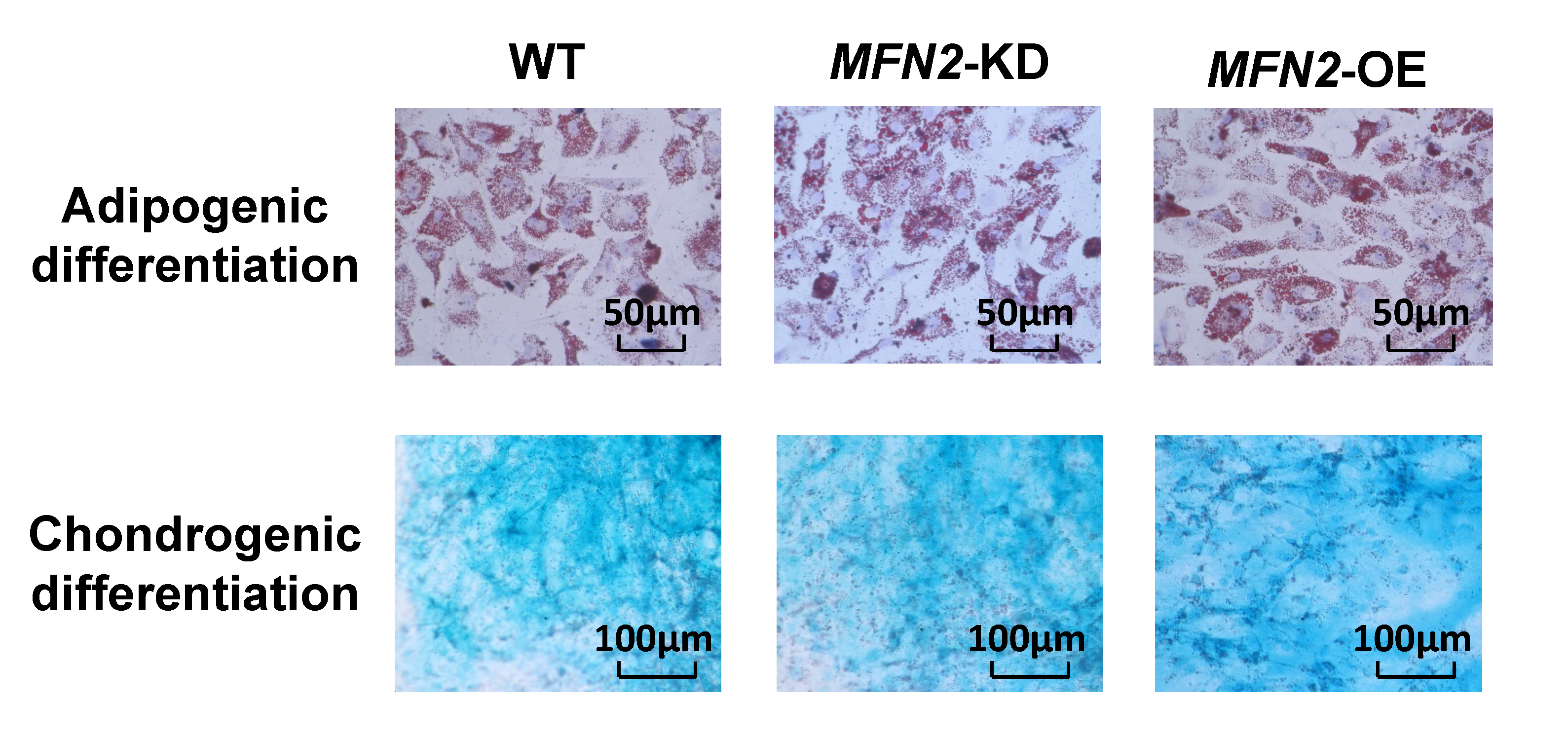

Supplement: Supplementary file 5 — Additional file 5: Fig. S5. Oil Red O staining and Alcian blue staining of WT, MFN2-KD and MFN2-OE iPSC-MSCs after adipogenic and chondrogenic differentiation. [file 13287_2022_2836_MOESM5_ESM.tif]

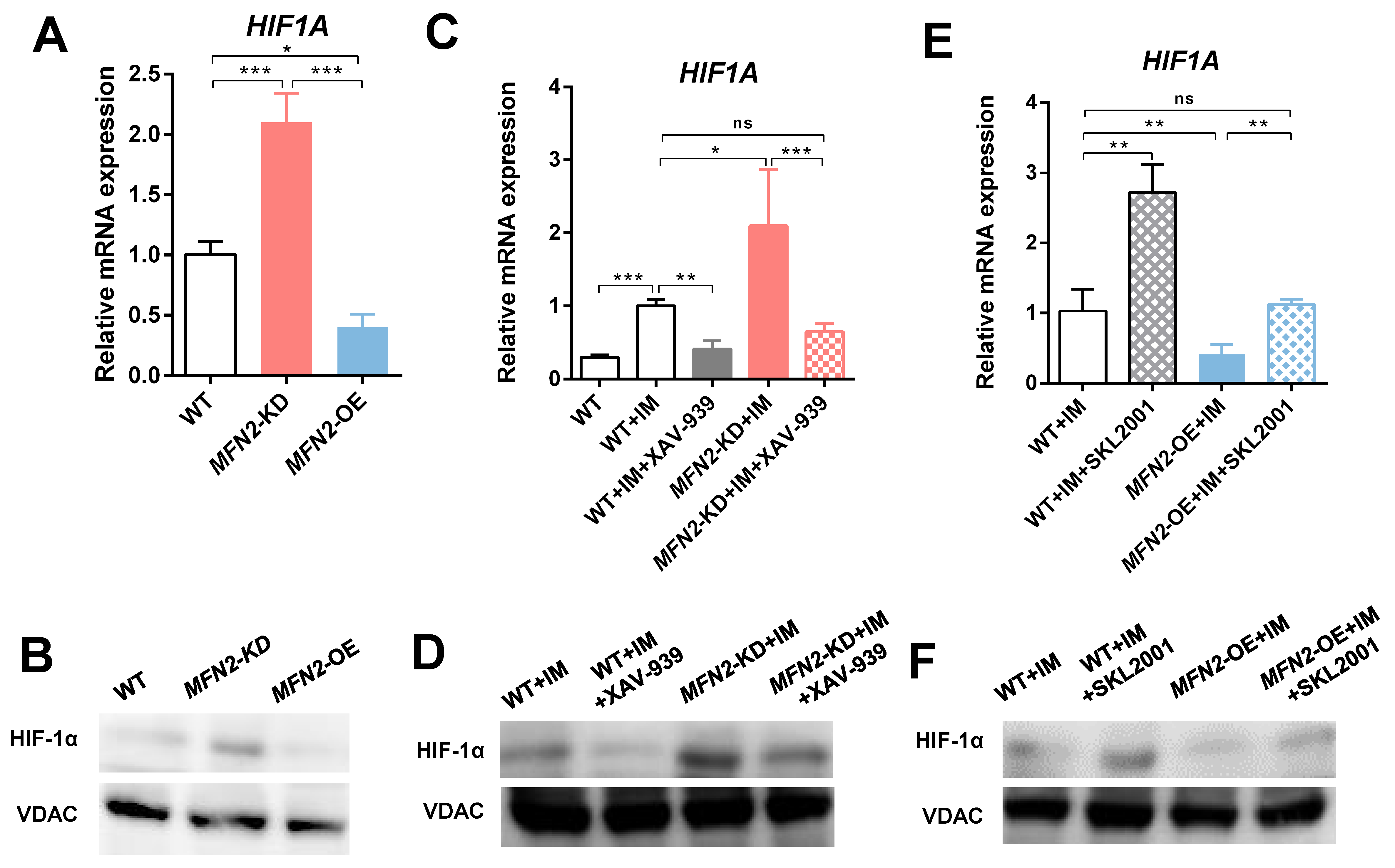

Supplement: Supplementary file 6 — Additional file 6: Fig. S6. qRT-PCR (A) and Western blot analysis (B) of HIF-1α in WT, MFN2-KD, MFN2-OE iPSC-MSCs. qRT-PCR (C, E) and Western blot analysis (D, F) of HIF-1α in WT, MFN2-KD, MFN2-OE iPSC-MSCs, and cells treated with XAV-939 and SKL2001 after osteogenic differentiation. [file 13287_2022_2836_MOESM6_ESM.tif]

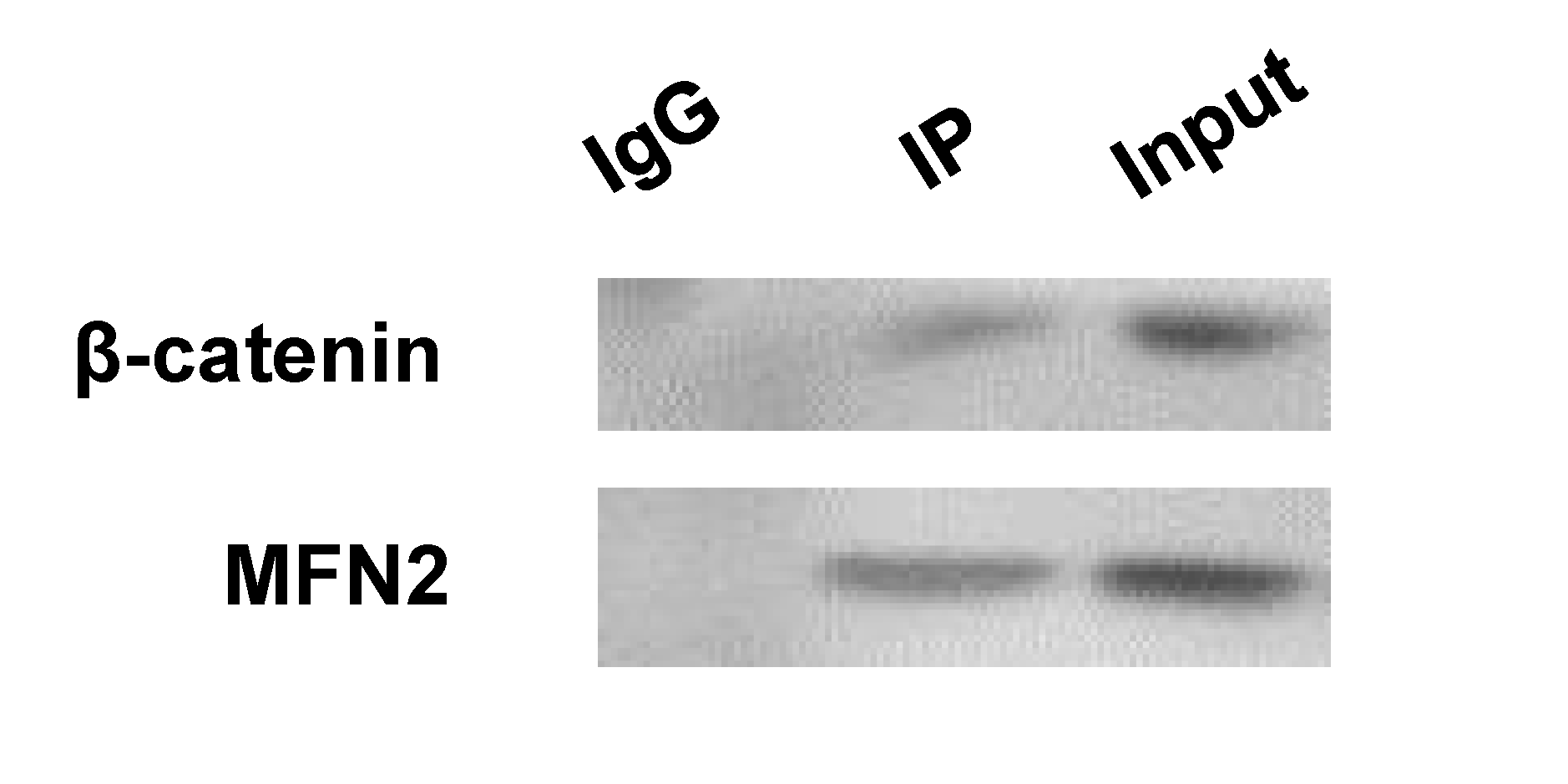

Supplement: Supplementary file 7 — Additional file 7: Fig. S7. Immunoprecipitation assay detected the binding relationship between MFN2 and β-catenin. [file 13287_2022_2836_MOESM7_ESM.tif]
